# ACTIVE problem solving: An action-oriented method for transforming Complex or Chaotic problems into verified reusable solutions

**DOI:** 10.1016/j.mex.2026.104165

**Published:** 2026-09-16

**Authors:** Reeder Vermaak, Meelan Roopa, Sara Grobbelaar

**Affiliations:** Department of Industrial Engineering, Stellenbosch University, Stellenbosch, South Africa

**Keywords:** Problem solving, Action-centered, Organizational knowledge, Cynefin, SECI, Dynamic capabilities

## Abstract

In problem landscapes characterized by extreme uncertainty, traditional analytical problem-solving often fails because causal relationships only become visible through intervention. Additionally, valuable insights obtained during crisis response frequently remain unarticulated and risk being lost after stability is restored if they are not deliberately recorded. This paper introduces ACTIVE (Action-Centered Transformation Into Verified Explications), a novel meta-methodology designed to bridge the gap between immediate crisis management and long-term organizational learning. By prioritizing action as a primary sensemaking tool, the framework allows practitioners navigating uncertain problem landscapes to convert ad-hoc solutions into verified, reusable dynamic capabilities.

The ACTIVE protocol guides the practitioner through the following process:

• Classifying the nature of the disorder to authorize experimental or rapid-response strategies where standard analysis is insufficient.

• Stabilizing the specific problem through targeted interventions that generate high-fidelity experiential data.

• Systematically abstracting the intervention into a generalized, explicit model to define its future validity.

By rigorously defining the problem structure post-intervention, ACTIVE allows organizations to transition problems from states of uncertainty to improved certainty or clarity. Ultimately, ACTIVE ensures that the exhaustive energy spent navigating chaos today is permanently converted into explicit, reliable efficiency for tomorrow.

## Graphical abstract

**Figure:**
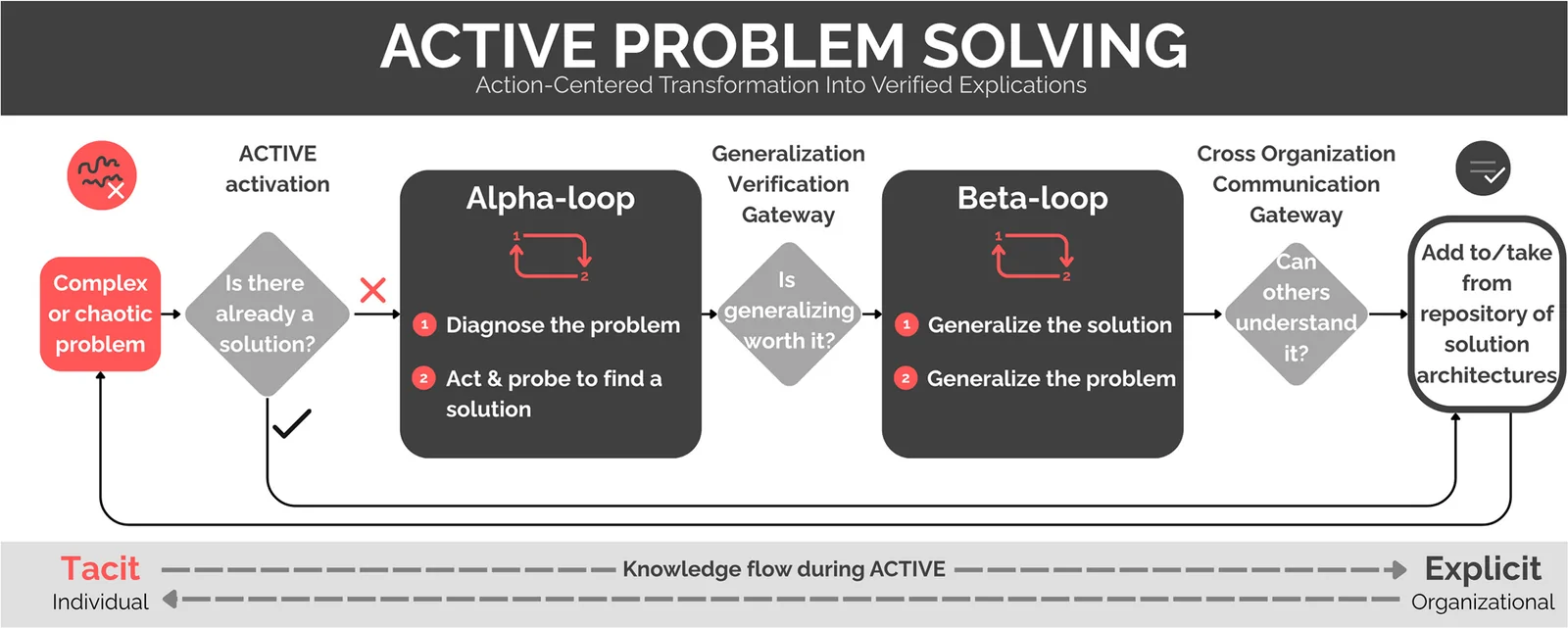

Specifications table

**Table:** 

**Subject area**	Engineering
**More specific subject area**	*Problem Solving, Crisis Management, Organizational Knowledge Generation, Decision-making theory*
**Name of your method**	*ACTIVE (Action-Centered Transformation into Verified Explications)*
**Name and reference of original method**	Cynefin [1], Nonaka - SECI [2], Dynamic Capabilities [3]
**Resource availability**	*Available upon request*

## Background

In his *Quotations* series, Cynefin Institute founder David Snowden laments that ‘the single most fundamental error of the last three decades is to try and design an idealized future state rather than working the evolutionary potential of the here and now’ [4]. In an era marked by rising uncertainty, effective problem solving has shifted from optimizing stable conditions to enabling agile decision-making in fluid problem landscapes [5]. This requires strategies that deliberately use action not merely as a consequence of a decision, but as an inherent feature of the decision process itself [6].

**Fig. 1:**
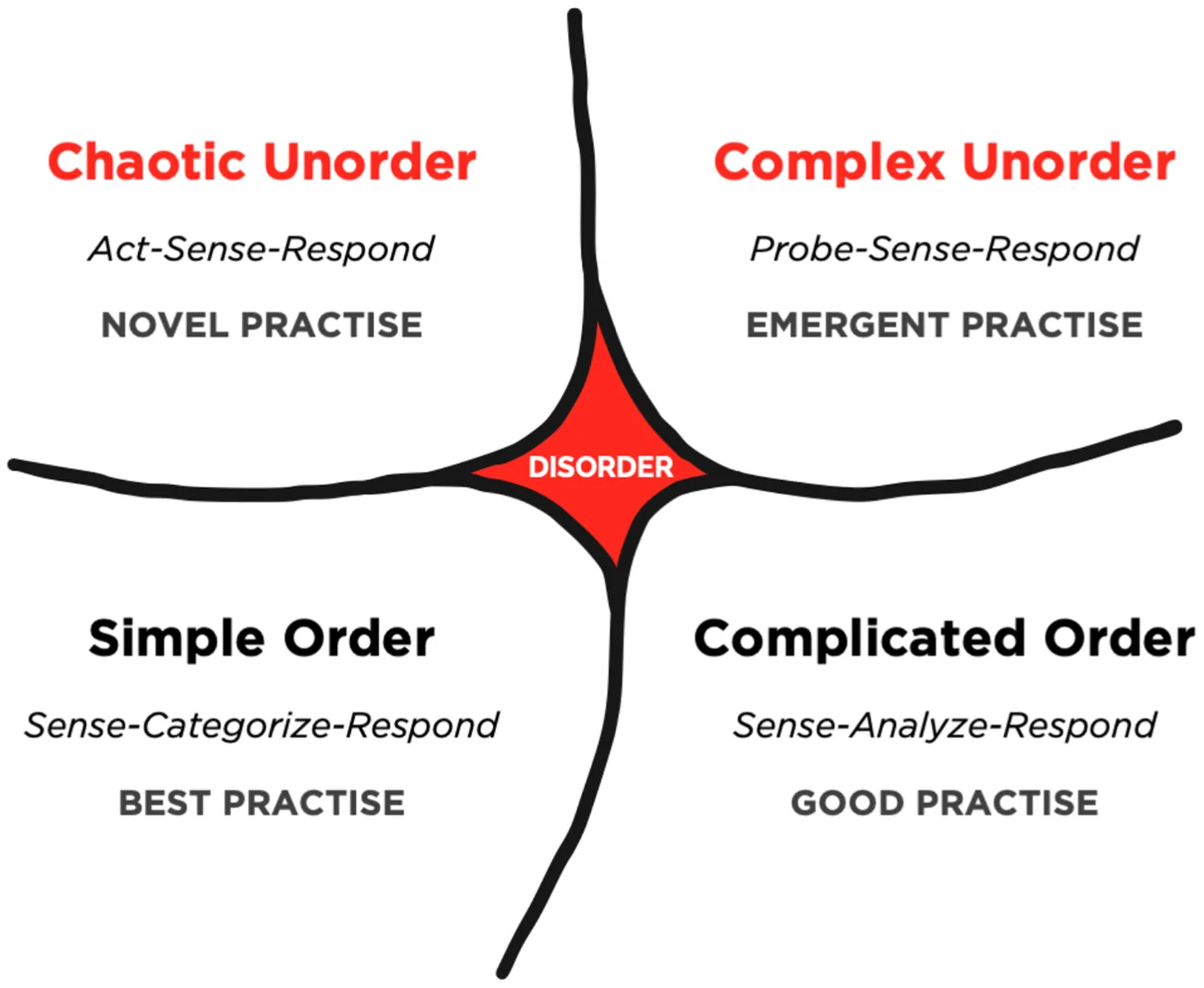
Cynefin Framework highlighting Ordered and Unordered problems adapted from Gorzeń-Mitka and Okręglicka [8].

While models such as Weick’s Sensemaking [7] or Brown’s *Design Thinking* [7] recognize that meaning evolves through interaction, the *Cynefin* framework most explicitly articulates how response strategies must match the problem context. As shown in Fig. 1, *Cynefin* classifies problems into "Ordered" domains (Simple, Complicated), in which cause-and-effect relationships are stable and analysis is effective, and "Unordered" domains (Complex, Chaotic) [1].

In Unordered contexts, clear causal patterns are absent or emergent, rendering upfront analysis insufficient [9]. Here, action becomes an essential mechanism for sensemaking. Consequently, this research focuses specifically on these Complex and Chaotic domains, where action-centered problem solving is necessary to preserve agility, clarity and confidence in the decision-making process [10].

However, agility alone is insufficient for long-term organizational resilience. As Nonaka [2] observes, in an economy where uncertainty is the only certainty, knowledge is the primary source of competitive advantage. Yet, the insight generated during these interventions is often tacit, highly subjective, and deeply rooted in the specific action taken. Unless this tacit knowledge is converted into explicit forms, it cannot be disseminated or leveraged by the organization as a whole.

Furthermore, as argued by Teece et al. [3], sustaining competitive advantage in rapidly changing environments requires development of dynamic capabilities, or the ability to integrate, build, and reconfigure a firm’s internal and external competences. While immediate agility may stabilize a crisis, strategic management theory emphasizes that adapting in a creative but disjointed way to a succession of crises does not constitute a dynamic capability [11]. To transform these fleeting, ad hoc interventions into true organizational capabilities, firms must actively apply cognitive effort to articulate and codify tacit experience into a learned and stable pattern of collective activity.

To address this, the study proposes ACTIVE (Action-Centered Transformation Into Verified Explications) problem solving. ACTIVE extends existing problem-solving strategies by ensuring that the insights gained during urgent interventions are not lost. Instead, it operationalizes deliberate learning to transform these ad-hoc solutions into verified generalizable methods, converting implicit action-based knowledge into explicit guidance for future use.

## Novelty

The primary procedural novelty of ACTIVE lies in its architecture as an integrative meta-method designed to manage and orchestrate pre-existing, standalone frameworks dependent on the exact operational context. Prior approaches such as Action Research [12] and Adaptive Management [13] provide localized, iterative feedback loops, yet they lack an overarching mechanism to transition contextually between disparate problem-solving modes. Similarly, retrospective tools such as After-Action Reviews (AAR) successfully isolate past lessons [14], and baseline Knowledge Management models map abstract tacit-to-explicit knowledge paths [2], but they remain disconnected from the real-time, exploratory toolkits such as Design Thinking [8], required to navigate highly unstructured settings.

Rather than introducing a competing tool, ACTIVE functions as a structural container that gives clear, non-linear structure to an ACTIVE practitioner navigating a volatile problem landscape through its double loops. Within this architecture, the ACTIVE practitioner has the immediate option to contextually deploy specific existing methods to solve the problem locally. Thereafter, if they verify the specific solution as effective and generalizable, they can deliberately transition the insight out of the Alpha Loop and generalize it into a permanent, reusable organizational asset. ACTIVE therefore bridges the gap between immediate resolving action and long-term capability building through a flexible, context-driven orchestration layer.

## Method details

Before the description of the method starts in earnest, the reader is advised to review Table 1 for a list of the standard definitions used across the method description. Furthermore, readers are encouraged to consult Appendix A, which provides an ACTIVE Protocol Flow diagram showing how a specific problem transitions through the double-loop architecture into a codified organizational asset.

**Table 1:** List of definitions used in ACTIVE.

Term	Definition	Core function
Problem Landscape	The specific set of Chaotic or Complex circumstances and fluid environmental events that make the **specific problem** initially unfamiliar, volatile, or impossible to understand through standard upfront analysis.	It defines the outer real-world environment where the **ACTIVE practitioner** must execute physical interventions to uncover hidden cause-and-effect relationships.
ACTIVE Practitioner	The decision-maker, researcher or team of individuals who are actively engaged with the **problem landscape**. They are directly responsible for creating a **specific solution** to the **specific problem** and translating their action-based experiences into generalized **organizational knowledge**.	The primary human agents navigating successive action-centered learning cycles to discover a verifiable solution.
Specific Problem	A highly localized, Complex or Chaotic crisis encountered within the **problem landscape** by the **ACTIVE practitioner**. Causal relationships within this problem are obscured, meaning it cannot be solved using traditional linear analysis or pre-existing standard operating procedures.	The immediate, real-world disorder that triggers the **ACTIVE practitioner** to initiate an action first intervention loop.
Specific Solution	The localized, action-based intervention constructed and executed by the **ACTIVE practitioner** to control, mitigate, and stabilize the **specific problem**.	A targeted experimental treatment (such as a prototype or triage action) designed to safely reduce operational chaos and generate high-fidelity learning data from the **problem landscape**.
Alpha Loop	The rapid, circular problem-solving space where the **ACTIVE practitioner** cycles continuously between discovering the interacting variables of the **specific problem** and testing a **specific solution**.	Representing the dynamic, high-velocity "learning-by-doing" phase where the **ACTIVE practitioner** relies on real-time feedback from the **problem landscape** to stabilize or better understand the **specific problem**.
Generalization Verification Gateway	A formal checkpoint where the **ACTIVE practitioner** evaluates whether the **specific solution** has successfully resolved the **specific problem** and if the proposed solution has merit for generalization into a **generalized solution**.	A strict quality-control gate requiring the **ACTIVE practitioner** to prove that the solution is effective, mature, and structurally sound enough before it is permitted to leave the **Alpha Loop** and enter the **Beta Loop**.
Individual Knowledge	The personal, highly subjective, and unarticulated tacit knowledge gained by the **ACTIVE practitioner** during their successive action cycles within the **problem landscape**.	The critical, hard-won operational insights that remain trapped inside the mind of the **ACTIVE practitioner** until they are deliberately externalized.
ACTIVE organization	The broader institution or corporate entity to which the **ACTIVE practitioner** belongs. It provides the overarching repository and systems required to absorb, store, and distribute verified knowledge assets to other teams.	The permanent structural home that ensures localized insights do not evaporate when individual personnel leave or when steady-state operations resume.
Beta Loop	The structured, strategic interaction space where the **ACTIVE practitioner** works with the **ACTIVE organization** to systematically formalize generalized **individual knowledge** into sustainable, long-term **organizational knowledge**.	The institutional learning loop where the relationship between a **General Problem** class and a **General Solution** architecture is permanently maintained and updated.
General solution	A generalized version or solution architecture derived from the verified **specific solution** built during the **Alpha Loop**.	A modular, decoupled operational template (such as a standardized routine or design pattern) that can be easily cloned, modified, and reused by the ACTIVE organization.
General problem	A systematically reframed and generalized classification of the **specific problem**, outlining the broader structural boundaries and context-specific conditions under which the **General Solution** can be safely applied.	A standardized problem typology that strips away superficial local symptoms to define the exact boundaries of future capability validity.
Organizational knowledge	The fully generalized, validated corporate memory stored systematically within the **ACTIVE organization**’s asset repository.	Reusable, institutional knowledge resources that can be instantly retrieved and used to inform, guide, and accelerate the response of future **ACTIVE practitioners** when they confront a similar crisis instance within the **problem landscape**.
Generalization	The deliberate, structured process occurring within the **Beta Loop** where the **ACTIVE practitioner** strips away the highly localized, physical details of a verified **specific solution** to extract its core, invariant principles.	To permanently convert tacit, action-based individual knowledge into an explicit, reusable organizational asset and map its broader validity boundaries within the **problem landscape**.
Cross-Organizational Communication Gateway	A formal checkpoint where the **ACTIVE practitioner** and organizational leadership evaluate whether the codified artifact is understandable, findable, and decoupled from localized jargon before it is published to the wider **ACTIVE organization**.	A communication quality gate that returns inadequately formatted artifacts to the **Beta Loop**, protecting the corporate repository from information bloat and low-fidelity documentation.

The primary objective of ACTIVE is to bridge the gap between immediate crisis management and long-term organizational learning. In high-uncertainty problem landscapes, organizations often rely on ad-hoc interventions to stabilize specific problems [10]. While momentarily effective, the knowledge generated during these interventions remains rooted in the intuition of the practitioner and the context of the event. This quickly proves unsustainable.

ACTIVE addresses this by formalizing the transition from intervention to explication. While this approach draws on Nonaka’s concept of converting tacit knowledge into explicit forms [2], and Zollo and Winter’s [11] notion of the Knowledge Evolution Cycle, ACTIVE distinguishes itself by operationalizing this process specifically for high-entropy problem landscapes where action must precede understanding. Nonaka’s framework relies heavily on *Socialization* and organizational redundancy to support knowledge creation organically over time [2]. In contrast, ACTIVE operates as a targeted interventionist framework, more in line with Zollo and Winter’s emphasis on routines as a method of developing dynamic capabilities in an organization [11]. It does not assume knowledge can be created through dialogue alone; instead, it mandates a structured *Externalization* phase immediately following stabilization. This ensures that the specific causal links revealed by the action are captured before the organization reverts to standard operations.

The ACTIVE framework operates on a double-loop logic. First, the Alpha Loop enables the ACTIVE practitioner to engage with the volatile problem landscape in a cyclical manner to iteratively address the specific problem with a specific solution. Once the practitioner reaches the milestone where they intend to transition this knowledge, they subject the specific solution to the Generalization Verification Gateway. This checkpoint evaluates whether the intervention, regardless of its localized operational effectiveness, contains stable, generalizable principles worthy of long-term preservation.

If the specific solution fails this gateway, it is dropped to protect corporate resource spend. If it passes, the Beta Loop commences. Within this loop, the practitioner, in collaboration with the ACTIVE organization, systematically defines the General Solution architecture and maps the structural characteristics of the corresponding General Problem class for storage within institutional knowledge systems. Once the Beta Loop completes, the implicit third loop of knowledge transfer can also be closed, as the individual knowledge of the practitioner has been generalized into organizational knowledge.

**Fig. 2:**
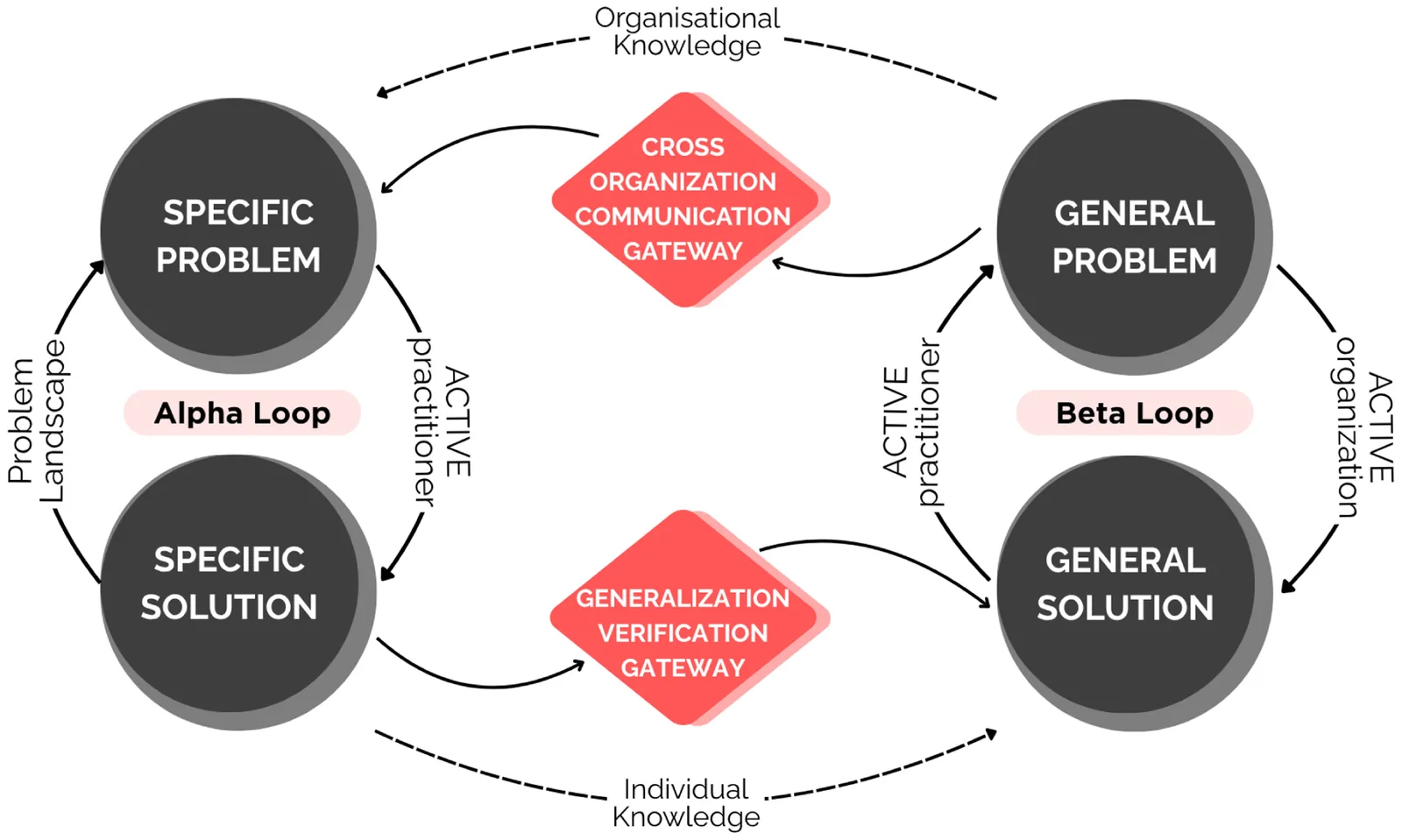
ACTIVE problem-solving methodology.

This asset can then be used by future practitioners to structure their Alpha Loop solutions and iteratively refine the generalized Beta Loop artifacts upon successive uses of the ACTIVE cycle for similar problems within the problem landscape. The entire ACTIVE cycle is visualized in Fig. 2.

## The problem lifecycle within ACTIVE

Before detailing the specific mechanics of the framework, it is necessary to understand how ACTIVE views the life cycle of a problem. Within this protocol, a problem is not a static entity; rather, it evolves from an unpredictable disorder into a structured operational asset. The life cycle begins in a Complex or Chaotic *Cynefin* domain*,* where a highly contextual crisis demands an immediate, action-first specific solution to generate feedback.

As the organization moves through the ACTIVE loops and gateway, the problem is stabilized, the tacit knowledge of the intervention is extracted, and the structural boundaries of the crisis are mapped. By the end of its lifecycle, the problem has been systematically transitioned into the *Complicated* domain. It is no longer a novel threat requiring leadership intuition, but a defined problem class matched with a verified, reusable solution.

### Alpha Loop

The initiation of ACTIVE begins when the ACTIVE practitioner actively senses and diagnoses a specific problem shifting within an unclear or volatile problem landscape. Since the problem landscape is characterized by uncertain cause-and-effect relationships as defined by the *Cynefin* framework, the specific problem cannot be understood in isolation from its surroundings. Instead, the practitioner must directly act within the problem landscape, triggering the Alpha Loop. Within this loop, the practitioner engages in a continuous, exploratory, or stabilizing action-based dialogue with the problem landscape.

The specific solution is not a predetermined blueprint brought from the outside; rather, it is dynamically co-created through the real-time collision and interaction between the practitioner's interventions and the landscape's feedback. This process is structured as a loop to reflect the highly cyclical nature of mapping un-understood environmental reactions [10]. The following section describes how the practitioner defines a specific problem as Complex or Chaotic according to the *Cynefin* framework. This is followed by a description of the tools and methods used to navigate this space, mapping the specific situational profiles where these tools are authorized. The section closes with the evaluation criteria used by the practitioner to decide if the specific problem has been sufficiently addressed and if the resulting specific solution should be advanced to the Generalization Verification Gateway.

### Alpha Loop – specific problems

The purpose of the Alpha Loop’s first stage is to diagnose the nature of uncertainty and validate whether an action-centered approach is required. In this stage, the practitioner "triages" the problem using the *Cynefin* framework, ensuring that ACTIVE is applied only where the limitations of traditional analytical approaches become apparent or are anticipated. The responsibility of deciding to classify a specific problem as either Complex or Chaotic and thus activating the deployment of ACTIVE sits squarely with the ACTIVE practitioner. Within ACTIVE, *Cynefin* is utilized as a sensemaking architecture to guide and focus the practitioner's exploratory approach rather than acting as a rigid classification tool. By avoiding hard-stop criteria for engagement, the framework allows the practitioner to dynamically navigate the fluid boundaries of the problem landscape, basing their operational decisions on real-time observation and adaptive sensing rather than prescriptive checklists.

ACTIVE is designed to work best for the Unordered domains of *Cynefin*, where causal relationships are obscured by complexity or turbulence [1]. Applying action-first strategies to simple or complicated problems, where stable best practices already exist, creates unnecessary risk. Therefore, Stage 1 is a filter that determines whether ACTIVE is an appropriate method.

ACTIVE is firstly appropriate in Complex contexts, characterized by flux, emergence, and competing hypotheses. Here, the goal of ACTIVE is to enable a “probe–sense–respond” strategy [1]. Examples include implementing new digital tools, navigating sustainability regulations, or merging distinct organizational cultures. In these scenarios, the interaction of multiple agents generates emergent effects that cannot be fully specified before intervention.

Secondly, ACTIVE is appropriate in Chaotic contexts, characterized by extreme instability and a complete absence of perceivable patterns. Here, the goal is to "act–sense–respond" to establish order [1]. Examples include active cybersecurity breaches, sudden supply chain shocks or leadership crises. In these moments, immediate action is required to restore minimal stability before sensemaking can begin.

By explicitly classifying the problem as Complex or Chaotic, Stage 1 prevents analysis paralysis [15]. It enables the organization to suspend the search for a perfect theoretical solution and proceed to Stage 2, where intervention generates the information necessary to resolve the problem. Thereby promoting more rapid decision-making.

To determine whether the deployment of ACTIVE is appropriate for a given situational configuration, the practitioner must evaluate the characteristics of both the problem and its ambient context. Practitioners can analyze the following diagnostic questions, shown in Table 2, to gauge if ACTIVE is an appropriate response to the specific problem facing them.

**Table 2:** Diagnostic questions to determine if ACTIVE is the appropriate response to a given specific problem.

Context	Goal of questions	Questions to ask
Indicators of an Unordered context (Complex or C")	These questions help the practitioner evaluate whether the specific problem has crossed the boundary from Ordered (Clear or Complicated) to Unordered (Complex or Chaotic) space, where linear causal loops are hidden and action must precede extensive analytical planning.	1. Are the cause and effect relationships within the problem landscape not understood or only recognizable in retrospect rather than predictable beforehand? [16]
		2. Do competing stakeholders default to highly fragmented or contradictory interpretations of the crisis, indicating that an objective, single right answer does not currently exist? [1]
		3. Have traditional, analytical problem-solving mechanisms or expert consensus repeatedly failed to resolve the issue, suggesting that standard reductionist checklists are no longer sufficient to stabilize the problem landscape? [1]
Indicators of a Complex Context (Requires Experimentation)	These questions assist the practitioner in identifying whether the problem landscape matches a Complex profile, meaning it possesses enabling constraints and can be gradually understood through small, safe-to-fail probes.	4. Is the problem landscape characterized by fluid, tightly coupled human interactions or shifting market dynamics where any localized tactical intervention causes unpredictable ripple effects across the system? [1]
		5. Can the practitioner design safe-to-fail experiments to reveal hidden patterns without risking the total operational collapse of the unit if an individual probe fails? [16]
		6. Does the specific problem require the practitioner to gather real-time feedback through a “probe-sense-respond” cycle before long-term resources can be safely allocated to a generalized version of the solution? [16]
Indicators of a Chaotic Context (Requires Immediate Stabilization)	These questions help the practitioner determine if the specific problem has entered a Chaotic state, where all constraints are broken, turbulence is total, and immediate authoritative intervention is required to establish basic order.	7. Is the speed of the crisis escalating so rapidly that any delay for analytical expert review or experimental probing will lead to immediate catastrophic failure? [1]
		8. Are cause-and-effect relationships completely non-existent at a structural level, requiring the practitioner to prioritize immediate operational stabilization over long-term root-cause analysis? [16]
		9. Must the practitioner adopt an “act-sense-respond” protocol by executing top-down commands first to create a temporary, stable boundary before any sensemaking can begin? [1]

### Alpha Loop – specific solution

Having diagnosed the nature of the disorder in Stage 1, the organization must transition from analysis to intervention. In the Specific Solution phase, ACTIVE operates as a container for action-centered methodologies, recognizing that in high-entropy problem landscapes, distinct causal relationships only become visible after an intervention has occurred. The goal is to generate the feedback necessary to understand the system’s interactions.

While classical procedural models lean heavily on deterministic checklists to enforce uniformity across different case interventions, this demand for upfront standardization is epistemologically counterproductive when navigating Complex or Chaotic problem landscapes [1]. In such landscapes, cause-and-effect relationships are non-linear and only perceptible in retrospect [16]. Prescribing tool selection upfront generates cognitive entrainment and confirmation bias, forcing practitioners to distort fluid organizational realities to fit rigid, predetermined structural criteria [17]. Therefore, ACTIVE intentionally rejects a deterministic checklist architecture in favor of a principles-based meta-framework. It relies on the practitioner's situation-dependent practical wisdom to select the appropriate tool or method to resolve the specific problem at hand [18].

When the problem is Complex, the objective is to learn faster than the problem landscape changes. Methodologies suitable for this context must support iterative learning [13], effectively treating every localized intervention as an experimental probe designed to surface latent system dynamics. To illustrate this procedural approach, ACTIVE highlights three prominent action-centered frameworks with broad applicability across diverse system environments: *Design Thinking* [7] for user-centric social complexity, the *Lean Startup* methodology [19] for market-driven validation, and *Adaptive Management* [20] for fluid socio-ecological or technical problems. These three frameworks provide complementary lenses for iterative sensemaking. They are treated as non-exhaustive examples. Ultimately, ACTIVE functions as an open, modular container; it remains entirely within the practitioner's situation-dependent judgment to evaluate if these specific choices are apt or if an alternative iterative problem-solving methodology is better matched to the unique parameters of their problem landscape.

For problems rooted in human behavior, unmet needs, or social dynamics, ACTIVE employs *Design Thinking*. As Brown articulates, this is not merely a styling exercise but a practice that leverages design methods to connect user needs with feasible technologies and sustainable business strategies that can realize value [7]. Within the ACTIVE protocol, *Design Thinking* is utilized to navigate the non-linear system of spaces rather than following a rigid set of orderly steps.

Teams use this method to uncover latent needs through direct observation and empathy, identifying constraints and opportunities that purely analytical methods might miss. ACTIVE leverages Brown’s concept that the goal of prototyping is not to create a finished product, but to evaluate the idea’s strengths and limitations and to uncover potential avenues for further development [7]. By generating tangible prototypes early, practitioners can elicit feedback from the situation, allowing the solution to evolve through user feedback before significant resources are committed.

When the complexity involves market viability or product strategy, ACTIVE utilizes the *Lean Startup* methodology. Ries [19] defines the core function of a startup as the creation of new products or services under conditions of extreme uncertainty. ACTIVE applies the Build-Measure-Learn feedback loop [19] to systematically test the unverified assumptions that underpin the initial solution approach inherent in the specific problem.

Instead of lengthy planning, teams are directed to build a Minimum Viable Product. As Ries emphasizes, this is the most efficient way to get through the Build-Measure-Learn feedback loop [19]. The specific solution generated in this phase is a rigorous demonstration that the team has discovered valuable truths about business prospects. This ensures that the organization does not merely execute a plan that leads nowhere but pivots or perseveres based on empirical evidence [21].

For complexity involving natural resources, technical systems, or large-scale operational environments, ACTIVE employs *Adaptive Management* [20]. This approach is essential when the impact of management decisions is uncertain and the system is dynamic, changing in response to both environmental conditions and the interventions themselves [20]. Williams and Brown [20] describe adaptive decision making as the practice of managing in a way that simultaneously drives outcomes and reveals the consequences of managerial decisions. In ACTIVE, operational interventions are treated as experimental treatments implemented according to a design brief.

This methodology provides a structured cycle of decision making, follow-up monitoring, and assessment. By comparing predictions generated by models against data-based estimates of actual responses, the organization reduces technical uncertainty over time, leading to improved management based on an accumulation of understanding about the problem and the solution strategy effectiveness [20]. Adaptive practices are especially important in high-risk, Complex environments, where formal policies lag behind unfolding structural crises.

**Fig. 3:**
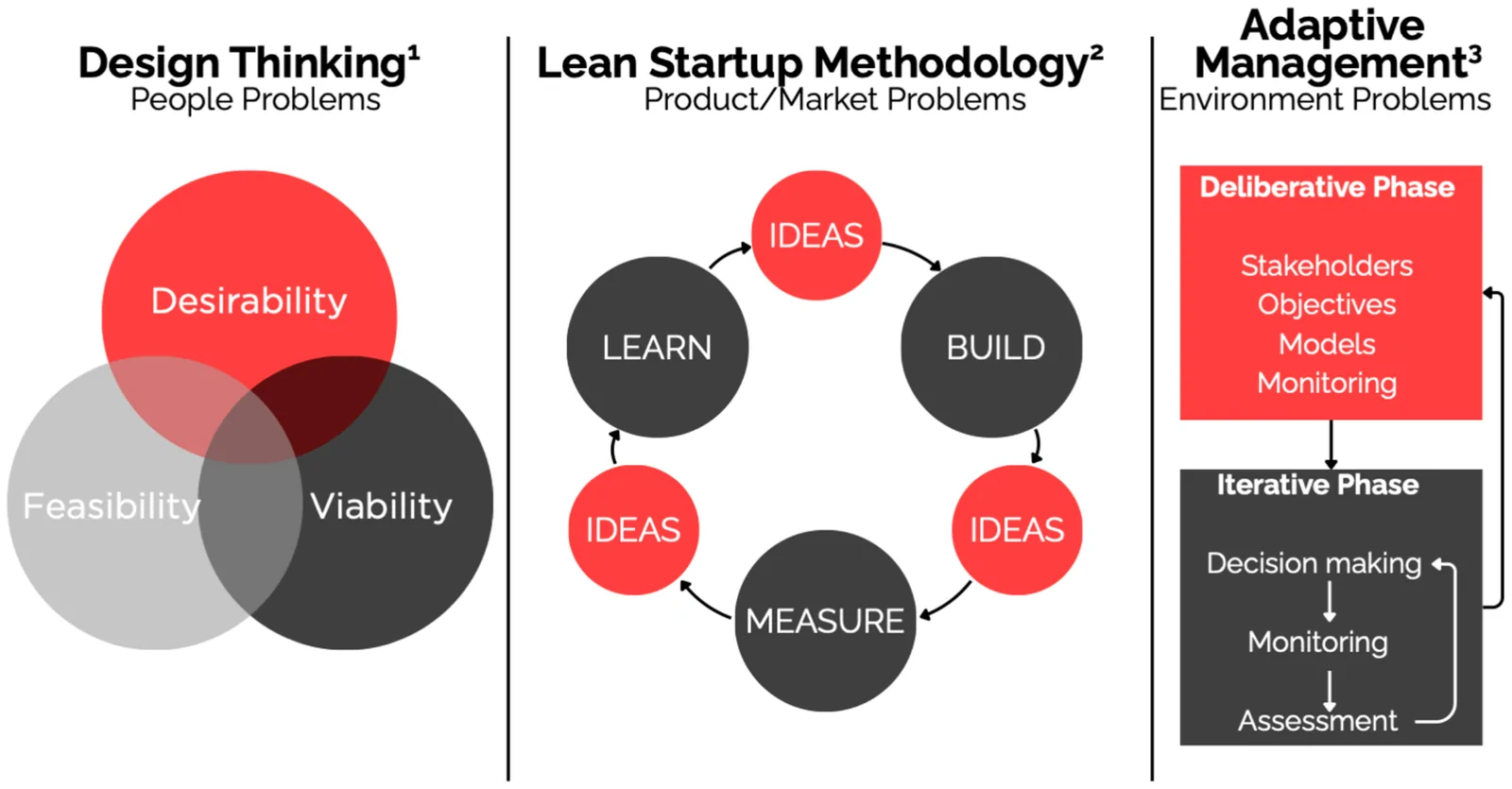
Action-centered problem-solving methodologies to address Complex problems. ^1^Design Thinking. Original redrawn interpretation from Brown (2008) [7]. ^2^Lean Startup Methodology. Original redrawn interpretation from Ries (2017) [19]. ^3^Adaptive management. Original redrawn interpretation from Williams and Brown (2014) [20].

What unifies these methodologies as applicable to Complex problems within ACTIVE is their shared reliance on deliberate action to surface system behaviors that cannot be known in advance. Fig. 3 shows the visualizations of the respective problem-solving approaches and the types of problems they can be most effectively applied to.

When the problem context is Chaotic, the luxury of iterative learning and hypothesis testing disappears. In these high-pressure scenarios, characterized by extreme instability and an absence of perceivable patterns, the immediate priority shifts from discovery to operational triage. To achieve rapid stabilization, ACTIVE highlights two primary baseline frameworks: *Recognition-Primed Decision Making* (RPD) [22] for situations demanding immediate, intuitive action under severe time stress, and the *CECA* (Critique-Explore-Compare-Adapt*)* loop [23] for structured, high-velocity adjustments. While these tools offer robust foundations for imposing order, ACTIVE does not mandate their use. Ultimately, it is left to the practitioner's situation-dependent judgment to evaluate the landscape and deploy whichever stabilization method or tool is best suited to bring immediate stability to the system.

For situations demanding immediate action under time stress, ACTIVE employs RPD [22]. Unlike analytical approaches that require the comparison of multiple options, RPD relies on the experience of the practitioner to classify a situation as typical or familiar [22]. By recognizing critical cues and causal factors, the practitioner can identify a plausible goal and a corresponding course of action without needing to generate large sets of alternatives.

ACTIVE utilizes RPD to bypass the paralysis of analysis [15]. Instead of optimizing, the protocol encourages selecting the first option that works rather than the best possible option. This is achieved through serial option evaluation, where a single course of action is assessed on its own merits rather than in comparison to others [22]. The feasibility of this action is tested through mental simulation, where the practitioner imagines the sequence of events to see if the plan will work or requires modification. This allows for rapid commitment to action while maintaining a check against potential failure.

To support dynamic decision making that goes beyond simple observation, ACTIVE incorporates the *CECA* (Critique–Explore–Compare–Adapt) *loop*. While traditional loops like *OODA* (Observe-Orient-Decide-Act) focus on observation, CECA places goal-oriented mental models at the center of the process to better handle the cognitive demands of command and control [23].

The process begins by critiquing the current state against a conceptual model of operations to identify information gaps, followed by active and passive data collection to update the situation model. The practitioner compares the current reality of the problem landscape against the conceptual model. When the reality invalidates the plan or blocks the path to the goal, the practitioner adapts the conceptual model and directs action to realign the system [23]. This framework provides the agility required in chaotic problem landscapes by ensuring that actions are driven by a coherent, evolving understanding of the crisis at play.

**Fig. 4:**
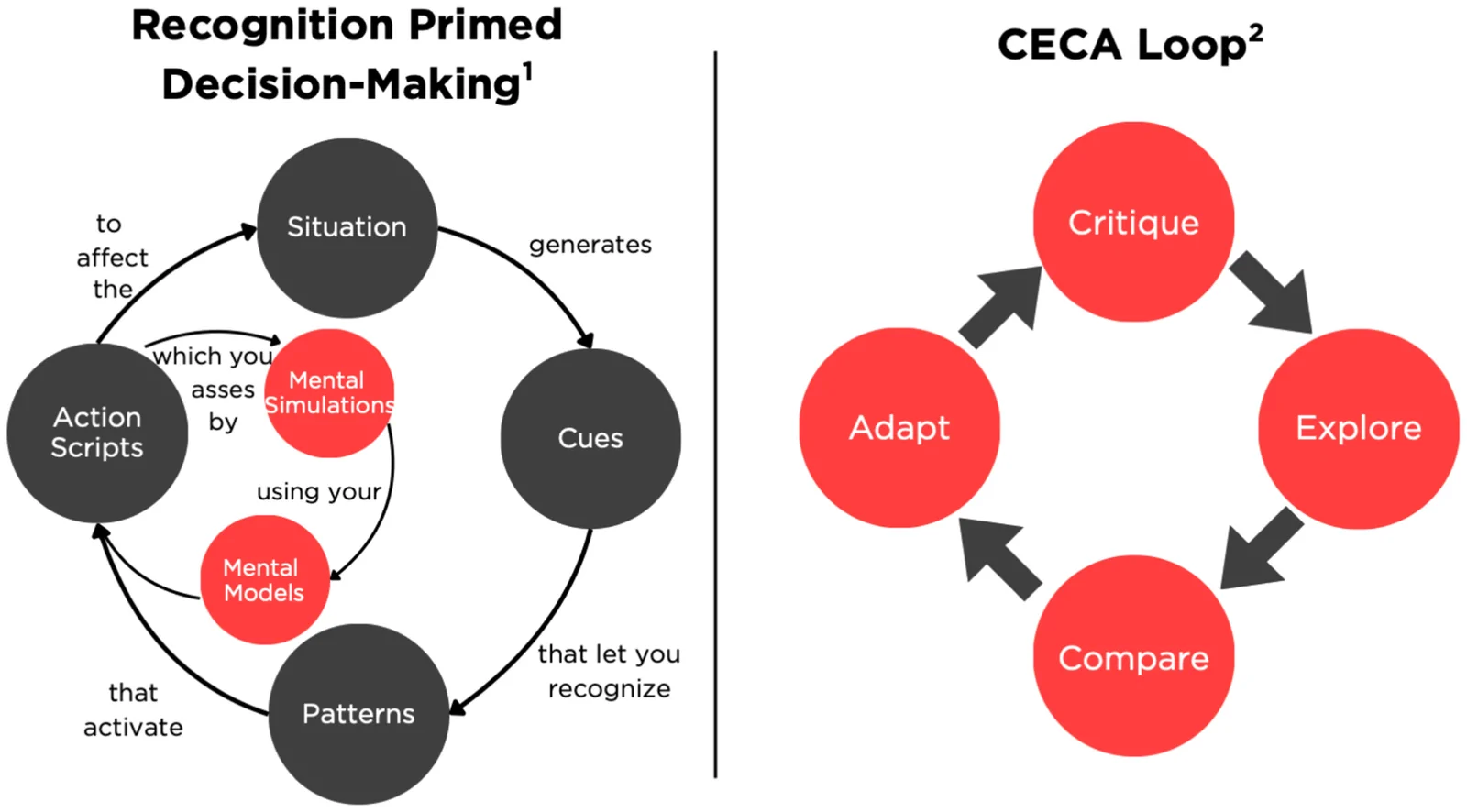
Action-centered problem-solving methodologies to address chaotic problems. ^1^Recognition primed decision making. Original redrawn interpretation from Klein (1995) [22]. ^2^CECA Loop. Original redrawn interpretation from Shawe and McAndrew [23].

Both RPD and the CECA loop are valued within ACTIVE for their capacity to restore a minimum level of stability and coherence. They do not seek to solve the problem permanently in one step, but to convert a chaotic context into a Complex one where further analysis becomes possible. Fig. 4 summarizes these action-centered methodologies applicable to Chaotic problem contexts.

Regardless of the specific tool selected, the output of Stage 2 is the same: experiential data. By taking action, the organization has generated a specific result, a stabilized situation, a working prototype, or a contained crisis. However, this success is currently trapped in tacit experience as it exists only as a specific event and the intuition of the people who solved it. To transform this solution into a lasting organizational asset, the process must move to Stage 3. Table 3 summarizes the application of the listed methodologies applied in ACTIVE, the minimum evidence thresholds needed to apply the methods, and their limitations.

**Table 3:** Summary of action-centered methodologies to solve Complex or Chaotic problems.

Methodology	Role within ACTIVE	Minimum Evidence Threshold	Key Limitations
Design Thinking [7]	Provides an action-oriented, empathy-driven way to probe complex human-centered systems through prototyping and feedback [7]	Complex problems involving human behavior, unmet needs, social or organizational dynamics [24]	Effectiveness depends heavily on the practitioner's ability to facilitate a situated conversation with the problem rather than following a linear process and adapting the tool to the specific context [25]
Lean Startup [19]	Structures rapid experimentation to test unverified assumptions via *Build–Measure–Learn* loops [19]	Complex problems involving market uncertainty, product–market fit, or strategic viability [26]	Possibility of skewed results resulting from cognitive biases or inadequate experimental rigor or in sectors with significant regulatory or safety hurdles [27]
Adaptive Management [20]	Treats interventions as experimental treatments to reduce uncertainty in dynamic technical or ecological systems [20]	Complex problems in operational, environmental, or large-scale technical systems, where the system is only partially visible and outcomes are dependent on variation and partial controllability [28]	Requires sustained monitoring capacity; learning cycles may be slow relative to crisis tempo [20]
Recognition Primed Decision Making [22]	Leverages expert intuition to rapidly categorize a situation based on cues and patterns, enabling the selection of the first workable course of action (satisficing) without comparing multiple options [22]	Chaotic or Time-Pressured Problems where immediate stabilization is required [22]	Relies heavily on expertise; vulnerable to cognitive bias if experience is limited or misapplied [29]
CECA Loop [23]	Implements a goal-oriented cognitive loop that prioritizes the critique of conceptual models against observed reality [23]	Chaotic problems requiring continuous adjustment rather than one-off action [23]	Cognitive load is high; effectiveness depends on clarity of conceptual models [23]

### Opening the feedback loop: generalization verification gateway

Once the ACTIVE practitioner brings the specific solution phase to a logical conclusion, a critical decision must be made: should the solution be proposed as a generalizable method to include in the organization’s knowledge repository, or does the analytical effort required to codify the practitioner's tacit knowledge represent a net loss of resources?

Before evaluating specific gateway questions to guide this decision, it is crucial to recognize that the ACTIVE methodology does not require an intervention to be perfectly successful to be deemed valuable. The core objective of ACTIVE’s generalization phase is to create reusable principles informed by both the successes and the failures experienced during the problem-solving process. In qualitative and case-based research methodologies, ineffective solutions or "failed replications" are treated as highly valuable sources of information used to explore the scope of a strategy [30]. By analyzing an ineffective solution, an ACTIVE practitioner establishes the boundary conditions of a theory or practice. Generalizing a failure allows the organization to explicitly map out where a specific approach is logically inconsistent or empirically impossible to use [30]. Furthermore, understanding the specific situational factors that led to a failure is a core component of valid case-to-case transfer, ensuring that future practitioners do not waste resources applying the wrong tool to a newly encountered problem [31].

With the value of both successful and failed interventions established, the practitioner must weigh the merits of generalization on a case-by-case basis. A primary caveat to consider is that ACTIVE functions as a meta-method; it is intended to be a flexible, overarching framework that supports rigor without constraining practitioner adaptation. Therefore, ACTIVE cannot mandate that an organization generalize all solutions, nor can it prescribe rigid, algorithmic criteria for what makes a solution generalizable.

Establishing strict criteria for the generalization of qualitative or case-based findings remains a contentious subject in academia, with methodologists noting a lack of explicit, universal rules for assessing when individual concepts are ready to be generalized [30,32]. Prescribing inflexible rules for generalization would only serve to artificially restrict solutions to those that conform to rigid parameters, potentially abandoning highly valuable edge cases to remain as fleeting, tacit insights.

Since generalizations derived from Complex problem landscapes often act as sensitizing concepts that provide flexible guidance rather than definitive, universal laws [31], the decision to generalize ultimately relies on the practitioner's judgment. The practitioner must evaluate the required investment of time and analytical effort against the solution's potential future utility. To assist in this evaluation, there are specific gateway questions the practitioner can use to illuminate whether the solution merits generalization, as shown in Table 4. However, while ACTIVE provides these questions to structure the decision-making process, it deliberately avoids dictating what the final decision must be, leaving the ultimate proposal of the solution up to the practitioner presenting their case to the ACTIVE organization.

**Table 4:** Generalization verification gateway questions.

Question	Justification
1. Recurring Vulnerability and Resource Worthiness	
Does the stabilized disorder represent a recurring class of problems where the long-term organizational value of preventing redundant experimentation outweighs the immediate analytical resources required to extract and codify the knowledge?	If the disruption was a once-in-a-generation anomaly, heavy analytical investment may be a net loss; however, if it exposes an ongoing operational bottleneck, the cost of generalization is justified to transform it into a reusable dynamic capability [33].
2. Principle-Driven Transferability	
Is the solution's effectiveness driven by invariant functional principles rather than highly localized, incidental contexts?	To be generalizable, the success of the intervention must rely on overarching systemic relationships or broad functional purposes rather than unique, unrepeatable flukes that cannot exist outside of the original specific event [31].
3. Proximal Similarity for Case-to-Case Transfer	
Are the material facts of the intervention (such as the actors, constraints, and social dynamics involved) proximally similar to what can be found in other units of the ACTIVE organization?	Valid case-to-case transfer relies on establishing gradients of similarity; a solution is only transferable if the practitioner can envision other people, settings, and times within the organization whose characteristics match the boundary conditions of the original crisis [31].
4. Structural Adaptability and Prototyping	
Can the core logic of the solution be successfully anonymized, stripped of its specific physical details, and generalized into an adaptable template?	A generalized asset must function as a "creational prototype" or sensitizing concept that future practitioners can clone, customize, and adapt to fit the nuances of newly encountered conflicts [31].
5. Knowledge Repository Novelty and Coevolution	
Has the organization’s existing knowledge repository been surveyed to ensure this solution is non-redundant, and if a similar method already exists, does this intervention provide crucial new adaptations?	Before generalization, the practitioner must confirm that the solution either adds a novel capability or updates an obsolete one, ensuring that organizational knowledge undergoes continuous coevolution rather than stagnating or duplicating existing tools [11].

Once the practitioner is confident in the generalization potential of their specific solution, they must present it as a candidate for organizational reuse to the ACTIVE organization’s leadership. The involvement of top management is crucial here, as major transitions within an existing business cannot succeed without astute strategic leadership [34]. Therefore, top leadership must actively evaluate the net value of the generalization effort and have the final say in allocating resources, moving the focus away from traditional operational management toward strategic organizational renewal.

If the practitioner is the leadership of the company, such as an SME owner, the situation requires additional discipline [34,35]. SMEs typically operate under severe resource constraints, limited managerial slack, and high environmental uncertainty, making the effective orchestration of resources both challenging and critical [35]. In these smaller problem landscapes, managerial intuition often dominates decision making. Consequently, SME owners must explicitly challenge their intuition-based decisions with evidence [35]. Before committing their highly constrained time and resources to generalizing a solution, these leaders should seek an impartial opinion or rely on a structured analytical foundation to verify their conclusions. This ensures that a measure of rigor is applied and that the value add truly justifies the potentially substantial spend of resources.

### Beta Loop

Once the generalization process receives formal authorization from leadership, the Beta Loop begins. This phase maps the collaborative interaction between the ACTIVE organization and the ACTIVE practitioner to generalize the specific problem and the specific solution into systematic, long-term capabilities. More precisely, the architecture outlines how the practitioner generalizes the tacit knowledge obtained during the localized intervention, explicating both the definitive blueprint of the General Solution and the structural characteristics of the associated General Problem class. Additionally, the practitioner explicitly maps the ambient boundary conditions of the problem landscape to dictate when the generalized asset remains valid. This structured data class is then archived within the organization's accessible knowledge management database. The cycle concludes as the practitioner and organization distribute these findings, informing subsequent practitioners of the updated repository so they can instantly check for available blueprints when encountering a new crisis within the problem landscape.

### Beta Loop – general solution

With the immediate problem addressed, the organization faces a critical window of opportunity. The specific solution exists, but it is currently trapped as tacit knowledge, highly personal, rooted in the intuition of the practitioners, and deeply embedded in the specific context of the event. If the process stops here, the organization has survived the incident but learned nothing from it.

Once the specific solution clears the Generalization Verification Gateway and leadership authorizes the necessary resource expenditure, the focus shifts entirely to executing the generalization process. Organizational learning research demonstrates that executing codification and generalization quickly after an intervention significantly reduces retrospective bias and knowledge decay [36]. To achieve this rapid translation, the framework suggests four distinct baseline pathways, allowing the ACTIVE practitioner to select the level of generalization that best fits the operational context. However, because ACTIVE operates strictly as a modular container of methods, the practitioner is under no obligation to utilize these default methods if they are aware of a more suited approach to their situation.

When the solution relies on human judgment or social dynamics, ACTIVE employs the *Externalization* mode of the *SECI model* [2]. Since tacit knowledge is often difficult to verbalize, this process does not demand immediate technical precision. Instead, it follows a natural cognitive progression:

1. **Metaphor:** The team first articulates what the solution felt like, using imagery to merge conflicting ideas.
2. **Analogy:** These contradictions are then reconciled by comparing the solution to known systems, bridging the gap between pure imagination and logical structure.
3. **Model:** Finally, the insight is crystallized into a transferable concept or logical structure that can be shared with the wider organization.

**Fig. 5:**
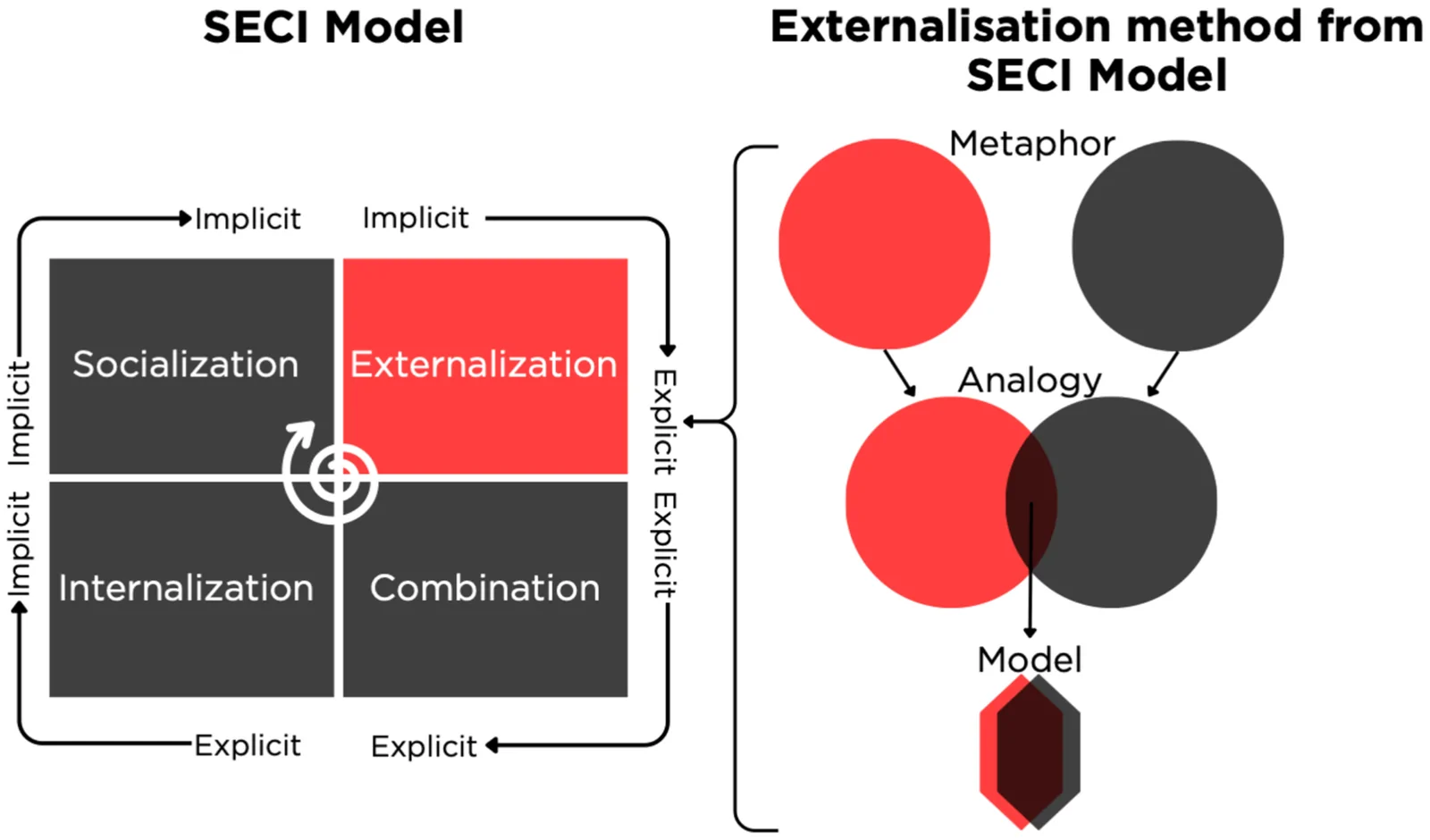
SECI model and externalization process. Original redrawn interpretation from Nonaka (1995) [2].

Fig. 5 visualizes the externalization phase of the SECI model.

**Fig. 6:**
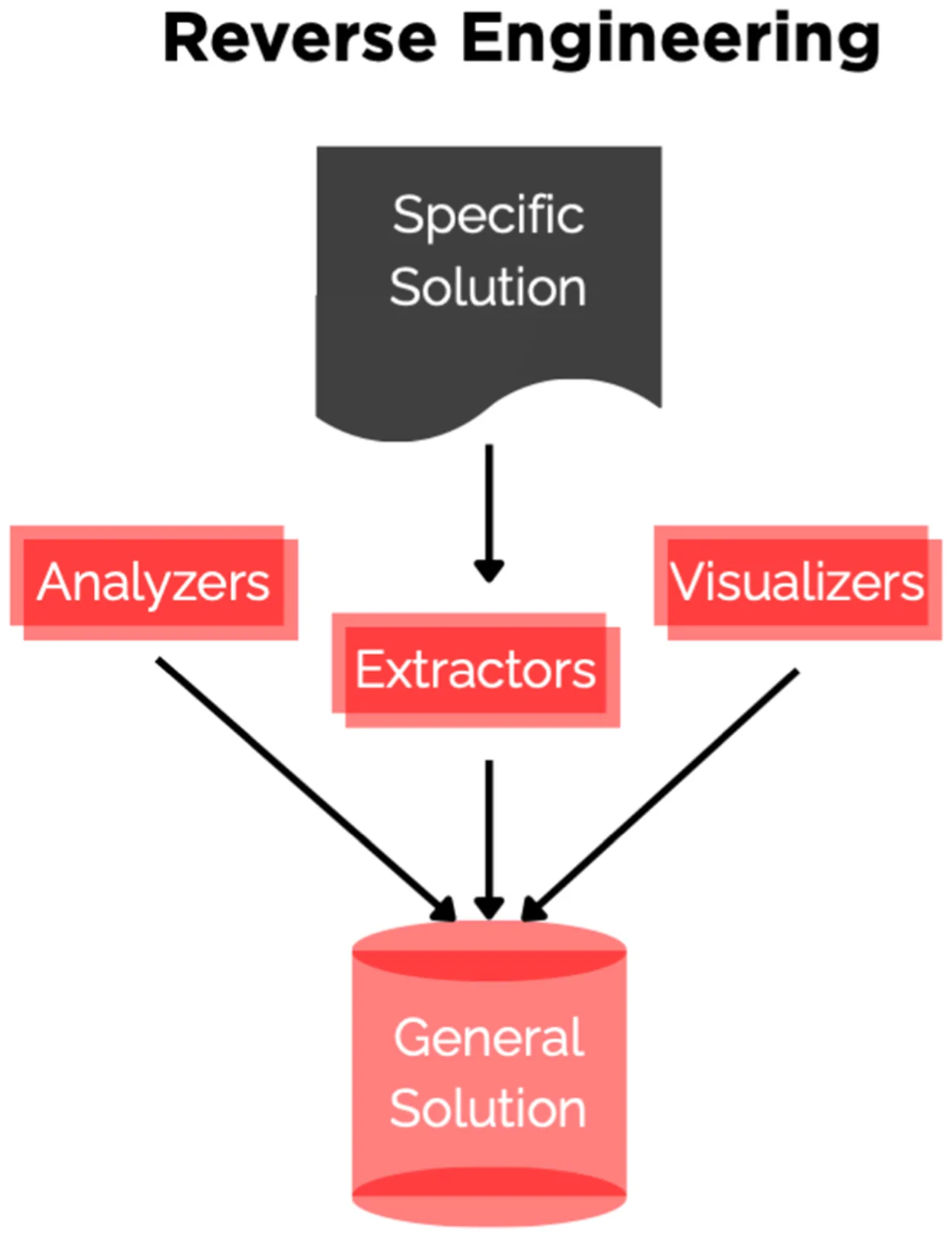
Reverse Engineering Principles. Original redrawn interpretation from Kienle and Müller (2010) [37].

When the solution is predominantly technical or mechanical, generalization through metaphor may be less central. Here, ACTIVE utilizes *Reverse Engineering* principles [37]. The focus shifts from "what were we thinking?" to "what actually happened?". By tracing the outcome backward to the structural elements and interactions that produced it, the team separates the essential success or failure factors from the incidental noise. This creates a rigorous structural blueprint of the solution, shown in Fig. 6.

For solutions that require flexibility rather than rigid standardization, ACTIVE utilizes *Prototypes* as a function of *Design Patterns* [38]. Rather than specifying a fixed rule, the successful intervention is captured as a concrete prototype: an adaptable template that future practitioners can clone and modify to fit new contexts. This approach preserves the core logic of the solution while acknowledging that the next iteration may require significant customization.

**Fig. 7:**
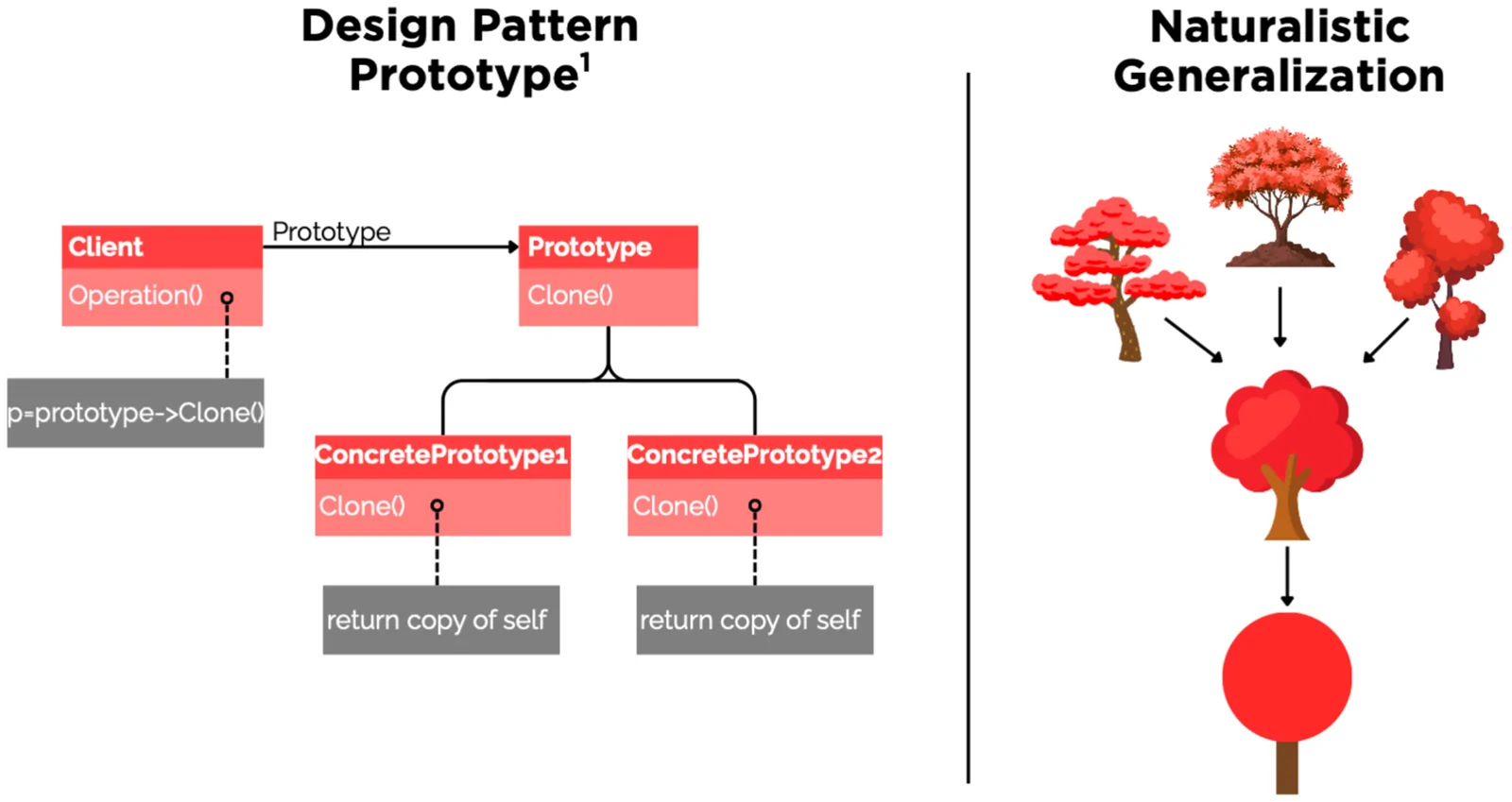
Simple solution generalization techniques. ^1^Design Pattern Prototype. Original redrawn interpretation from Gamma et al. (2011) [38].

Naturalistic generalization provides a complementary, cognitively lightweight mechanism for generalization by leveraging the experiential judgment of practitioners [39]. Through structured reflection on the resolved case and recognition of similarities with prior experience, practitioners derive heuristics that link technical insights to lived practice. Although less formal, this approach is particularly valuable in maintaining agility, as it enables rapid recognition and response without requiring full methodological reconstruction. Fig. 7 visualizes these simpler generalization techniques.

The output of Stage 3, regardless of the pathway chosen, is a verified, generalized method which has been stripped of its specific problem context and formatted for reuse. Table 5 summarizes the methodologies useful for generalizing the specific solution, the minimum evidence threshold to justify the use of one of the listed methodologies and key limitations. A solution is, however, only effective when applied in the correct context. Therefore, the final step of ACTIVE is to define the boundaries of this solution in Stage 4.

**Table 5:** Role of solution generalization methods within ACTIVE.

Methodology	Role within ACTIVE	Minimum Evidence Threshold	Key limitations
SECI Externalization (Metaphor → Analogy → Model) [2]	Converts tacit insight into explicit, shareable conceptual models by articulating a vision of the world. Uses metaphors to bridge the gap between experience and language, followed by analogies to resolve contradictions, finally crystallizing into an artifact [2]	Solutions grounded in human judgment, teamwork, social interaction, or where interpretive knowledge is primary	Metaphors can remain ambiguous if not disciplined into models, and require high personal commitment from the individual to articulate the tacit knowledge [2]
Reverse Engineering [37]	Reconstructs causal structure of a successful intervention from observable outcomes to structural elements [40,41]	Technical, mechanical, or process-driven solutions where the system logic is obscured but discoverable	Could require high cost and complexity for extractors and visualizers to be effective [40]
Creational Design Patterns (Prototype) [38]	Captures solutions as adaptable templates rather than rigid rules. Allows future practitioners to clone the logic while modifying the implementation [38]	Situations where flexibility and reuse matter more than standardization	Solutions cannot be reused unchanged; effective reuse depends on the practitioner's ability to understand the context and adapt the prototype to fit new constraints [38]
Naturalistic Generalization [39]	Enables rapid generalization through experiential reflection and pattern recognition [39]	Time-sensitive problem landscapes or expert-driven domains	Low formal rigor; difficult to audit or transfer across expertise levels and disregards assumptions and limitations for the cost of generalization

### Beta Loop – general problem

The effectiveness of the ACTIVE protocol relies on the understanding that a solution is only valid within a specific context. As Schön [42] articulates, professionals in real-world practice do not simply solve given problems; they must construct them from messes, characterized by uncertainty and disorder. The initial diagnosis and action in the Alpha Loop was merely a frame imposed on the situation to make it manageable. Having generalized the solution in the first part of the Beta Loop, the practitioner and the organization face a final choice: which analytical lens best maps the boundaries within which to apply this new knowledge? ACTIVE does not prescribe a rigid sequence here. Instead, it provides a flexible set of generalization methods, from which the practitioner selects the method that best reveals the problem's hidden structure.

Action often reveals that the initial diagnosis of a problem was superficial. Reframing is useful in scenarios where symptoms of the problem masked the true cause [42]. In this case, the intervention executed in Stage 2 will have generated feedback from the situation, revealing unintended effects and new phenomena that challenge the initial understanding. By reflecting on this transactional relationship between the action taken and the system's response, practitioners can reconstruct the problem definition to align with the reality encountered during the intervention [42]. This ensures that the General Problem definition captures the systemic nature of the challenge rather than just the superficial symptoms that triggered the initial response.

In complex technical or organizational systems, it is often difficult to distinguish between the essential functions of a solution and its incidental physical details. Here, ACTIVE practitioners can employ Work Domain Analysis, specifically utilizing Rasmussen’s *Abstraction Hierarchy* [43]. This method allows the organization to model the system across multiple levels, distinguishing between the physical implementation of the solution and its functional purpose.

The analysis moves bottom-up, starting with the *Physical Form* (the specific resources or tools used in the Specific Solution) and the *Physical Functions* (the specific processes employed). The process then abstracts these elements into *Generalized Functions* (standard processes or feedback loops independent of specific equipment) and finally to the *Functional Purpose* (the high-level system objectives and constraints) [43].

**Fig. 8:**
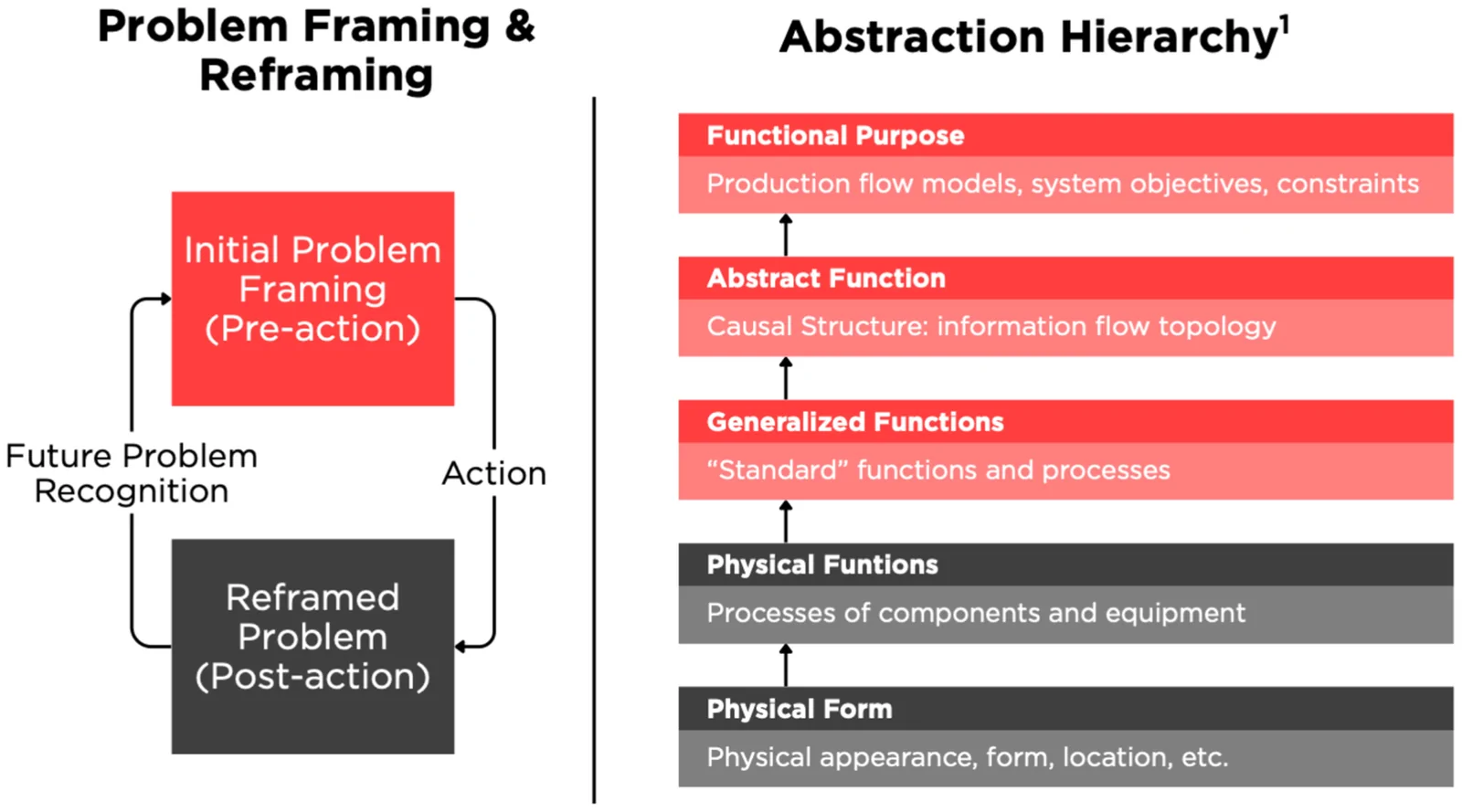
Sense-making methodologies for Complex problem generalization. ^1^Abstraction hierarchy. Original redrawn interpretation from Rasmussen(1986) [43].

By mapping the problem along the reframing and generalizing principles shown in Fig. 8, the organization identifies the invariant causal links that made the solution effective. This creates a map that allows future practitioners to recognize when the same functional problem has arisen, even if the physical indicators look different or begin to vary over time.

While the *Abstraction Hierarchy* maps the vertical structure of the problem, *General Morphological Analysis* can be used to map the lateral possibilities and non-quantifiable relationships within the problem context. When a solution’s success depends on a specific combination of variables, problem generalization can be based on Zwicky’s method for investigating *Totality* [44]. This step involves identifying the essential parameters (dimensions) of the problem and the spectrum of possible conditions for each.

ACTIVE utilizes *General Morphological Analysis* to construct a morphological field that creates a typology of the problem context. Crucially, the team performs a *Cross-Consistency Assessment* to evaluate pair-wise relationships between conditions [45]. This process reduces the total set of theoretically possible configurations to a smaller "solution space" of internally consistent scenarios. This helps the organization identify where the current solution applies and where it would be logically inconsistent or empirically impossible to use it.

Finally, should a solution work specifically because it leverages or bypasses a bottleneck, *Constraint Analysis* can prove useful to explicitly define the problem’s boundaries and validity conditions. Goldratt posits that every system is limited by at least one constraint that prevents it from achieving higher performance [46]. In Stage 4, the organization uses Effect-Cause-Effect logic to validate the root cause of the disorder that necessitated the intervention. The analysis distinguishes between physical constraints (bottlenecks in resources) and policy constraints (rules or mindsets that limit performance) [46].

To resolve conflicts discovered during the intervention, the protocol employs the *Evaporating Clouds* method [46]. This technique does not seek compromise but attempts to invalidate the conflict by exposing the hidden assumptions behind the opposing requirements.

**Fig. 9:**
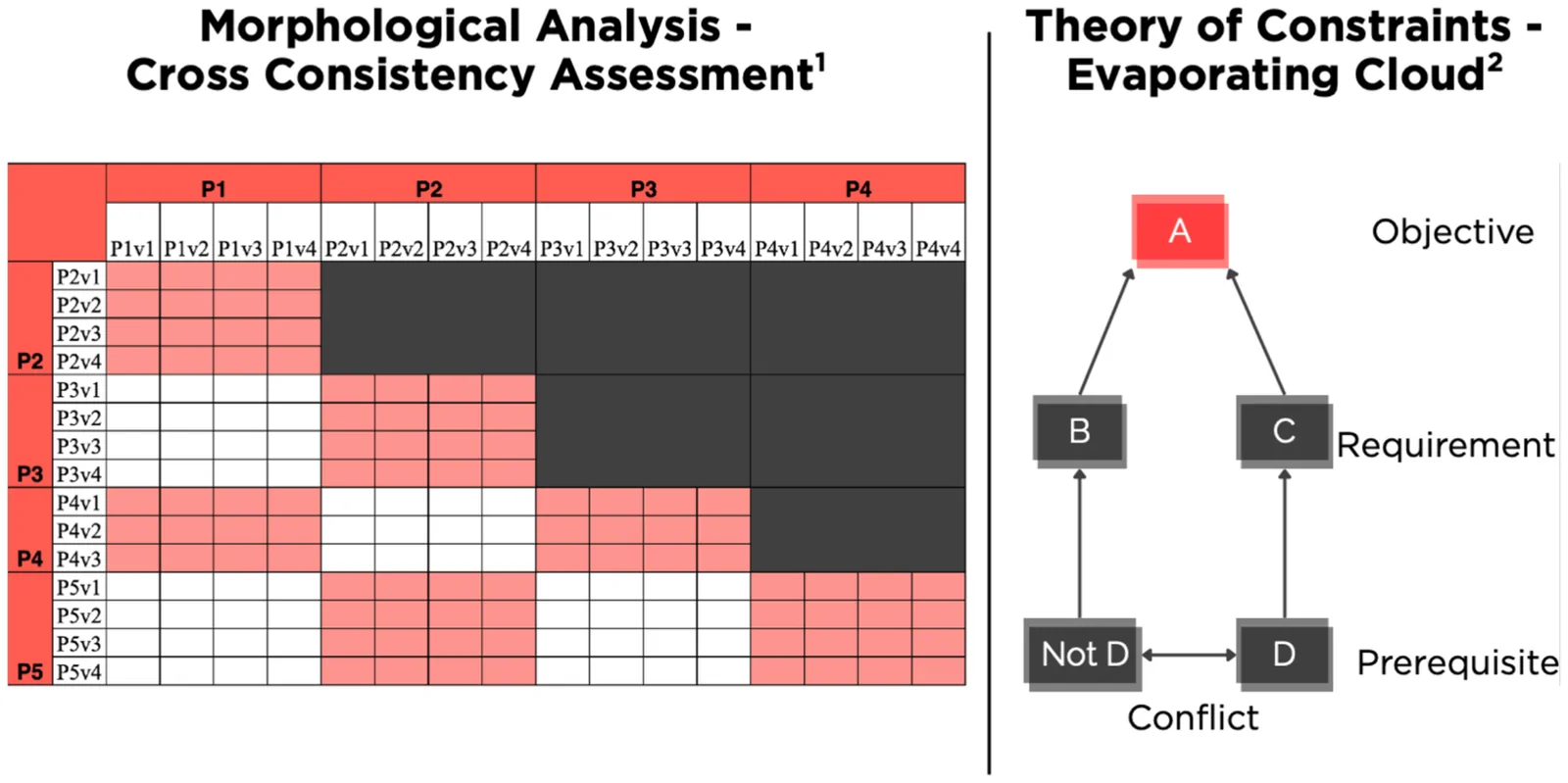
Possibility-space clarification methodologies for Complex problem generalization. ^1^Cross Consistency Assessment. Original redrawn interpretation from Ritchey (2015) [47]. ^2^Evaporating Cloud Method. Original redrawn interpretation from Goldratt (1990) [46].

Fig. 9 illustrates how Morphological Analysis and the Theory of Constraints work to clarify the possibility space and the limitations of the General Problem.

At its core, the Beta Loop is designed to mitigate long-term complexity. This is achieved by anchoring problem structures and defining the exact boundary conditions of a General Solution, theoretically allowing a class of problems to shift from *Cynefin*'s Unordered domains to the Complicated domain. Nevertheless, in alignment with the fluid realities of complex systems, this domain transition must be evaluated with strict caution. An absolute shift into a predictable state is an ideal target rather than a guaranteed end-state, as dynamic problem landscapes frequently cause structured routines to slide back toward chaos. To counter this decay, ACTIVE anchors its value in organizational learning. Formally logging these verified pathways into corporate memory acts as an adaptive reconfiguration routine. This directly reflects the microfoundations of Dynamic Capabilities, transforming perishable individual insights into a systemically reconfigurable knowledge asset capable of absorbing persistent environmental shocks [3].

In this new state, the problem is no longer a crisis requiring leadership intuition; it is an understood problem class, with a verified, standard operating procedure. This closes the ACTIVE loop: the confusion of the first attempt becomes the clarity of established practice. Table 6 summarizes the role of each problem generalization methodology within ACTIVE, the minimum evidence thresholds to decide to use one of the listed methodologies and their key limitations.

**Table 6:** Summary of problem generalization methodologies in ACTIVE.

Methodology	Role within ACTIVE	Minimum Evidence Threshold	Key limitations
Problem Framing & Reframing [42]	Reconstructs what problem was actually solved, post-intervention [42]	Problems where symptoms initially masked deeper structural causes	Subjective; depends on reflective capacity of practitioners
Work Domain Analysis (Abstraction Hierarchy) [43]	Identifies invariant functional relationships underlying the solution [43]	Complex technical or organizational systems	Requires analytical effort and modeling expertise
General Morphological Analysis [44]	Maps the full configuration space to define where the solution applies [45]	Problems with many interacting variables and non-quantifiable relationships	Can become complex, resource-intensive and encourage binary reasoning
Theory of constraints [46]	Explicitly defines bottlenecks that governed solution effectiveness [46]	Problems dominated by physical or policy constraints	Risk of oversimplification and polar framing where multiple interacting constraints exist in actuality

### Closing the feedback loop: cross-organizational communication gateway

Once a General Solution and its problem boundary conditions have been codified in the Beta Loop, the framework mandates a final formal checkpoint before the asset is permanently deployed: the Cross-Organizational Communication Gateway. While the Generalization Verification Gateway evaluates whether a solution merits codification, this gateway evaluates whether the resulting explicit artifact is sufficiently understandable, accessible, and decoupled from localized jargon for the rest of the organization to reference and implement.

The decision to pass an asset through this gateway relies heavily on the ACTIVE practitioner's situational judgment to evaluate communication readiness, combined with organizational leadership's strategic authorization to manage the corporate knowledge repository. Rather than enforcing rigid, algorithmic documentation checklists, this gateway utilizes a principles-based evaluation to ensure the artifact is an effective "sensitizing concept" across diverse corporate units. To assist in this evaluation, the practitioner and leadership analyze the five diagnostic questions outlined in Table 7.

**Table 7:** Cross-organizational communication gateway questions.

a	Justification
1. Semantic Decoupling and Comprehensibility	
Is the artifact stripped of highly localized acronyms, team-specific jargon, and context-dependent noise so that a practitioner in an entirely different business unit can immediately comprehend its core logic?	For explicit knowledge to be usable by the wider organization, it must be crystallized into a standard conceptual model or shared language. If an asset remains trapped in the local dialect of the originating team, it fails to achieve cross-functional utility [2].
2. Architectural Scannability and "Clonability"	
Is the General Solution structured as an open, modular template (such as an adaptable routine or design pattern) that future teams can quickly scan, clone, and customize without needing to consult the original authors?	Reusable corporate assets should function as "creational prototypes" rather than rigid, step-by-step instructions. High scannability reduces cognitive load and prevents information friction during cross-organizational knowledge transmission [38].
3. Operational Findability and Metadata Alignment	
Has the artifact been assigned clear, standardized problem typologies and contextual tags so that it can be instantly retrieved when a future practitioner searches the repository during a crisis?	An organization’s capacity to rapidly reconfigure its competencies relies heavily on the findability of its knowledge resources. If future teams cannot systematically search and uncover a historical match during a crisis, the organization experiences costly, redundant experimentation [3].
4. Absorptive Capacity and Training Readiness	
Does the broader ACTIVE organization possess the requisite baseline knowledge (absorptive capacity) to safely interpret and deploy this protocol, or does its distribution require targeted, high-velocity briefing mechanisms?	Organizational learning requires that the receiving unit has the cognitive capacity to absorb and utilize the transferred asset. Assessing readiness mitigates the risk that complex or volatile frameworks are dangerously misapplied by untrained teams [48].
5. Coevolutionary Feedback Mechanisms	
Does the logged asset contain explicit, built-in loops that prompt future practitioners to log their own localized variations, performance metrics, and adjustments upon reuse?	Dynamic capabilities emerge from the continuous coevolution of codified guidelines and shifting real-world experience. The asset must function as a living document that evolves with every successive environmental shock [11].

### Managing the double-loop lifecycle

If the artifact fails to satisfy these communication benchmarks, it is returned to the Beta Loop for formatting refinement to protect the corporate repository from information bloat and low-fidelity documentation. If it passes, top leadership formally authorizes its publication to the central knowledge management database.

Once published, the cross-organizational feedback loop is officially established. When a future practitioner encounters a new crisis within the fluid problem landscape, their first step is to execute an inward sensing search of this repository. Instead of treating every Complex disruption as an entirely novel event, they leverage these path-dependent, historically verified solutions to instantly stabilize new uncertainties, adjusting the protocol to fit their unique operational reality and closing the macro-learning loop of the ACTIVE organization.

## Boundary conditions: where ACTIVE should not be applied

ACTIVE is deliberately narrow in scope, and its usefulness depends on that narrowness. It should not be used where cause and effect are already understood, since Simple and Complicated problems are better served by established best practice or by expert analysis [1]. It should also be avoided wherever a first action cannot be made safely. In landscapes carrying severe safety, legal, or regulatory exposure, no probe is genuinely safe-to-fail, and a failed intervention costs the organization more than the learning it returns. A third exclusion is organizational rather than technical. Where an organization lacks the reflective capacity or the repository needed to absorb what it learns, only the Alpha Loop will ever run, and the Beta Loop will never close [48]. ACTIVE then degrades into unstructured firefighting. Finally, a genuinely non-recurring event seldom justifies the analytical cost of generalization, and the Generalization Verification Gateway is designed to reject such cases [31].

### Limitations

While ACTIVE provides a structured framework for converting uncertainty into actionable, reusable knowledge, it is not without limitations. By design, ACTIVE emphasizes action as the starting point for sensemaking and learning, followed by systematic generalization of both solution and problem characteristics. However, this approach introduces several constraints that practitioners should consider to ensure its effective and responsible application.

ACTIVE depends critically on the practitioner correctly identifying and classifying the problem type as Complex or Chaotic. Misclassifying a Simple or Complicated problem and applying an action-first approach could lead to inefficient use of resources or suboptimal outcomes. Care must be taken to ensure that ACTIVE is applied only in contexts where the problem exhibits high uncertainty, unclear causal relationships, or emergent behavior, as recommended by the *Cynefin* framework. Incorrect application undermines both the efficiency and validity of the method.

The effectiveness of ACTIVE is influenced by the tools and methods chosen at each stage, as well as the skill and judgment of the practitioner in applying them. Because each problem, organization, and practitioner is unique, results are inherently context-dependent and not fully replicable. ACTIVE is therefore best understood as a flexible, overarching guide that offers structured suggestions rather than a prescriptive or formulaic solution. Practitioners must exercise judgment in selecting appropriate methodologies for problem framing, action, and generalization. Leaning on a multidisciplinary approach within the organization can assist in informing better judgment.

ACTIVE is intentionally broad and not tailored to a specific industry, scope, or regulatory problem landscape. While this flexibility is a strength, it also opens the door for cognitive biases to influence decisions, particularly during the specific problem stage or when using tools such as *Recognition-Primed Decision Making, CECA Loop*, or *Naturalistic Generalization*, which rely on expert judgment. ACTIVE mitigates bias through the methodological generalization of both problem and solution: by capturing contextual criteria and generalizing solution strategies, practitioners and organizations can refine and improve methods over repeated applications, progressively reducing the influence of individual biases.

Although ACTIVE aims to reduce uncertainty in future problem-solving, it requires an initial investment of time and effort to execute action, capture tacit knowledge, and generalize solution strategies. Organizations facing extreme time pressure or resource constraints may find the method challenging to implement fully. Furthermore, the iterative nature of ACTIVE means that optimal benefits, such as transferable generalized methods and reduced future complexity, emerge only after repeated applications and careful reflection.

### Case study scenario

As part of the method validation, the ACTIVE problem-solving method will now be applied to a hypothetical case study scenario to illustrate its use. This case study scenario is limited in scope and only utilizes a few of the tools and methods available to a practitioner in a qualitative manner.

### Problem landscape

A local manufacturing plant servicing a domestic market relies on a single class of raw materials imported from a foreign supplier. A sudden tariff-based geopolitical shock introduces immediate cost increases, uncertain lead times, and contractual ambiguity. The causal relationships among procurement costs, inventory policies, and customer demand are obscured by external environmental volatility.

### Alpha Loop – specific problem identification

**Fig. 10:**
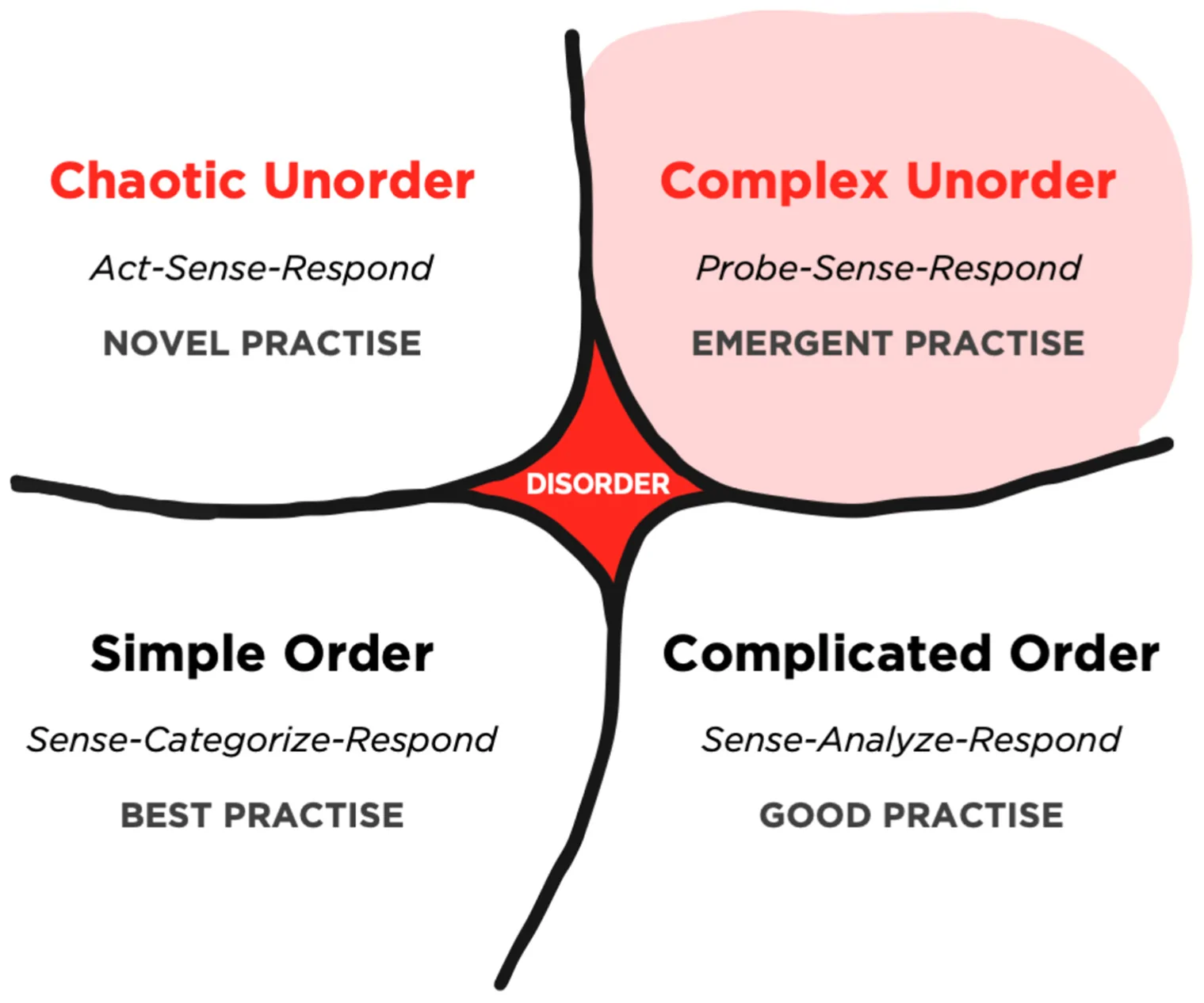
Cynefin classification of specific problem.

In the wake of a sudden tariff shock, the lack of visibility into procurement and customer demand relationships leads the ACTIVE practitioner to classify the problem within the *Cynefin* framework's Complex domain, highlighted in red in Fig. 10. Since dynamic variables interact too unpredictably to permit traditional upfront planning [1], the practitioner deploys the Alpha Loop to utilize action for exploratory sensemaking. This shifts the operational focus away from predicting a definitive answer toward a continuous, iterative interaction with the problem landscape using safe-to-fail probes to prompt legible system behaviors.

### Alpha Loop – specific solution generation as a continuous feedback loop

Once inside the Alpha Loop, the practitioner deploys *Lean Startup’s* Build-Measure-Learn cycle [19] to drive a high-velocity feedback loop. Given that the problem is deeply rooted in market uncertainty and strategic business viability, this framework is an appropriate operating vehicle. It ensures that the emergent specific solution is an intervention dynamically co-created through real-time collisions with the problem landscape.

**Fig. 11:**
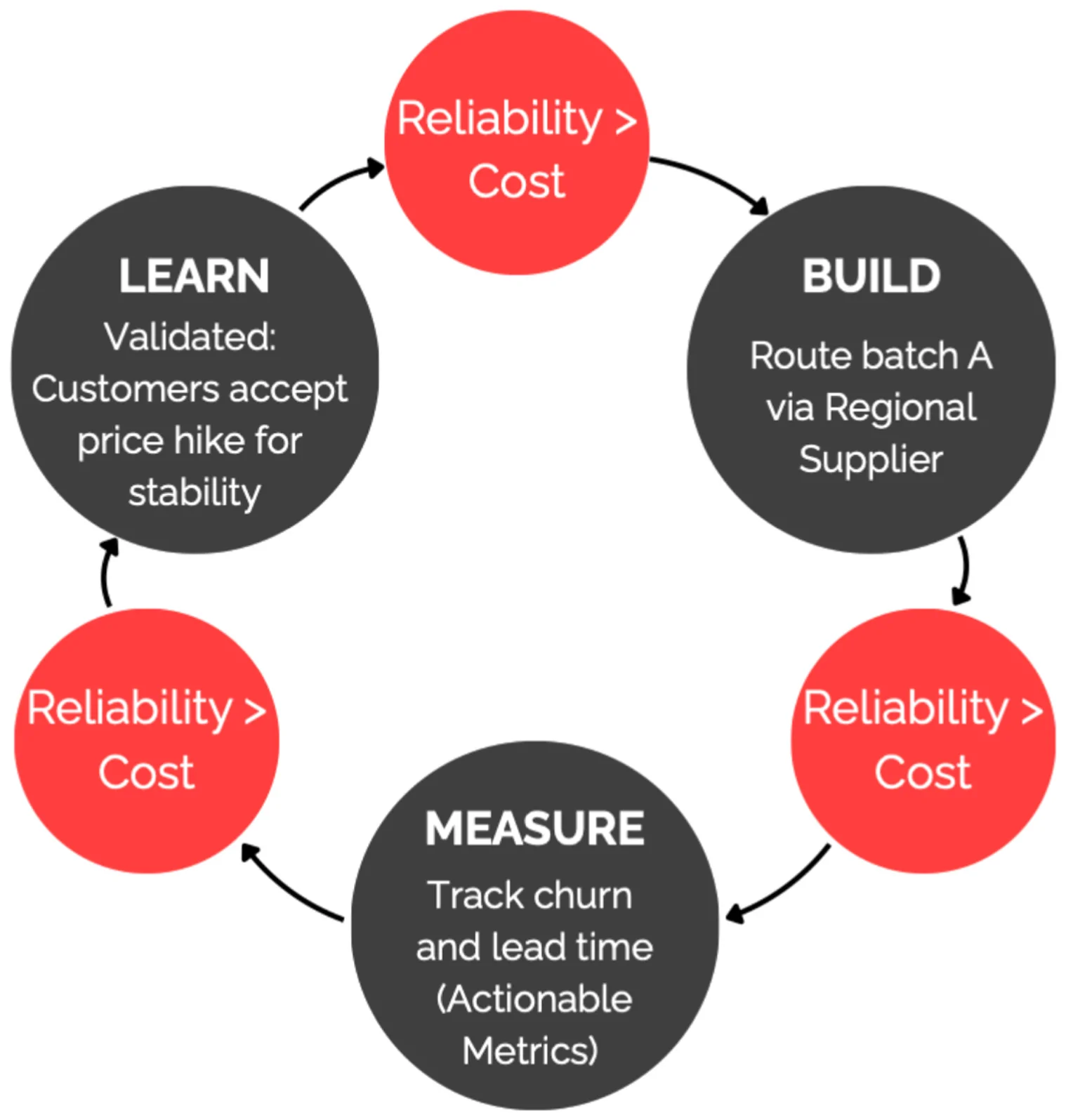
Build-Measure-Learn feedback loop from Lean-Startup Methodology.
• **Cycling the Loop:** Armed with this adjusted problem definition, the practitioner continues the loop. They iterate on the specific solution, expanding the hybrid sourcing contracts to more product lines based on the new data, and measure the landscape's reaction again. The practitioner cycles continuously in this learning-by-doing phase, constantly mapping the un-understood environmental reactions by toggling between discovering the true variables of the specific problem and testing the specific solution against them.
• **The Transition:** Reaching a Resolved Space. After several successive action cycles within this rapid loop, the practitioner recognizes that the high-reliability/high-cost supply channel is successfully and consistently mitigating the crisis. The immediate operational chaos has been safely controlled, and the practitioner assesses that the specific problem has been brought into a stabilized, "resolved space".

• **The Initial Probe:** The practitioner executes a Minimum Viable Supply Strategy by routing a small, high-cost batch of materials through a secondary regional supplier. This physical intervention is injected directly into the problem landscape to test a core assumption: that customers will prioritize reliability over cost.
• **Landscape Feedback and Problem Adjustment:** This tactical action directly impacts the problem landscape, and the landscape immediately feeds empirical data back to the practitioner: customer churn remains at 0 % despite a notable unit cost increase. This crucial feedback directly adjusts the practitioner's understanding of the specific problem and is shown in Fig. 11. It is no longer viewed merely as a cost optimization crisis, but is dynamically reframed within the loop as a delivery predictability challenge.

At this logical milestone, the practitioner stops to reflect on the experiential data generated by the intervention. Because the specific solution has proven structurally sound and highly effective in controlling the disorder, the practitioner realizes that the hard-won, tacit knowledge generated during this continuous loop contains stable, generalizable principles. Concluding that this solution might have strong generalization potential to help other units facing similar supply shocks, the practitioner officially concludes the Alpha Loop and advances the solution to the Generalization Verification Gateway to formally evaluate its long-term merit.

### Generalization verification gateway

With the immediate operational chaos safely controlled and the problem landscape brought into a stabilized space, the ACTIVE practitioner pauses to reflect on the specific solution. Before committing the organization's analytical resources to codify this tacit knowledge, the practitioner must systematically evaluate the intervention against the five diagnostic questions of the Generalization Verification Gateway.

By subjecting the high-reliability/high-cost supply channel to this gateway, the practitioner provides the following simple validatory answers to confirm its generalization potential:

1. **Recurring Vulnerability and Resource Worthiness:** Does the stabilized disorder represent a recurring class of problems where the long-term organizational value of preventing redundant experimentation outweighs the immediate analytical resources required to extract and codify the knowledge?
  **Yes.** Geopolitical shocks and supplier disruptions represent ongoing, systemic vulnerabilities for the manufacturing plant. The long-term value of having a rapid-response capability easily outweighs the short-term cost of generalizing the knowledge.
2. **Principle-Driven Transferability:** Is the solution's effectiveness driven by invariant functional principles rather than highly localized, incidental contexts?
  ***Yes.*** *The solution worked because of the invariant functional principle that customers will prioritize delivery predictability over cost during high market turbulence, rather than relying on a lucky fluke tied only to this specific foreign tariff.*
3. **Proximal Similarity for Case-to-Case Transfer:** Are the material facts of the intervention proximally similar to what can be found in other units of the ACTIVE organization?
  ***Yes.*** *Other manufacturing units and product lines within the broader organization share similar external supplier constraints, logistics networks, and domestic customer dynamics, making case-to-case transfer highly viable.*
4. **Structural Adaptability and Prototyping:** Can the core logic of the solution be successfully anonymized, stripped of its specific physical details, and generalized into an adaptable template?
  ***Yes.*** *The specific supplier contracts and material classifications can be easily stripped away, leaving behind the core strategic logic of intentionally substituting efficiency for buffered redundancy when volatility spikes.*
5. **Knowledge Repository Novelty and Coevolution:** Has the organization’s existing knowledge repository been surveyed to ensure this solution is non-redundant, and if a similar method already exists, does this intervention provide crucial new adaptations?
  ***Yes.*** *A survey of the corporate repository shows it currently only contains standard Just-in-Time cost-optimization protocols. This intervention adds a completely novel, volatility-responsive dynamic capability that the organization previously lacked.*

Having successfully validated all five criteria, the practitioner formally concludes that the action-based insights contain stable, generalizable principles. They then present this specific solution to the ACTIVE organization’s top leadership, who evaluate its strategic value, formally authorize the resource expenditure, and officially transition the problem out of the Alpha Loop and into the Beta Loop.

### Beta Loop – a dialogue between the ACTIVE practitioner and the ACTIVE organization

With the specific solution successfully validated and informal commitment secured, the problem transitions into the Beta Loop. This phase represents a structured, strategic interaction space where the ACTIVE practitioner and the ACTIVE organization collaborate to formalize the tacit insights from the intervention into sustainable, long-term organizational knowledge.

This translation is inherently a dialogue. The practitioner brings the lived, action-based experience of stabilizing the crisis, while the organization represents the broader institutional context. Together, they must converse and negotiate how to extract the core logic of the intervention so that the resulting generalized solution integrates into existing organizational practices and achieves true strategic fit.

### Beta Loop – generalization of solution principles

**Fig. 12:**
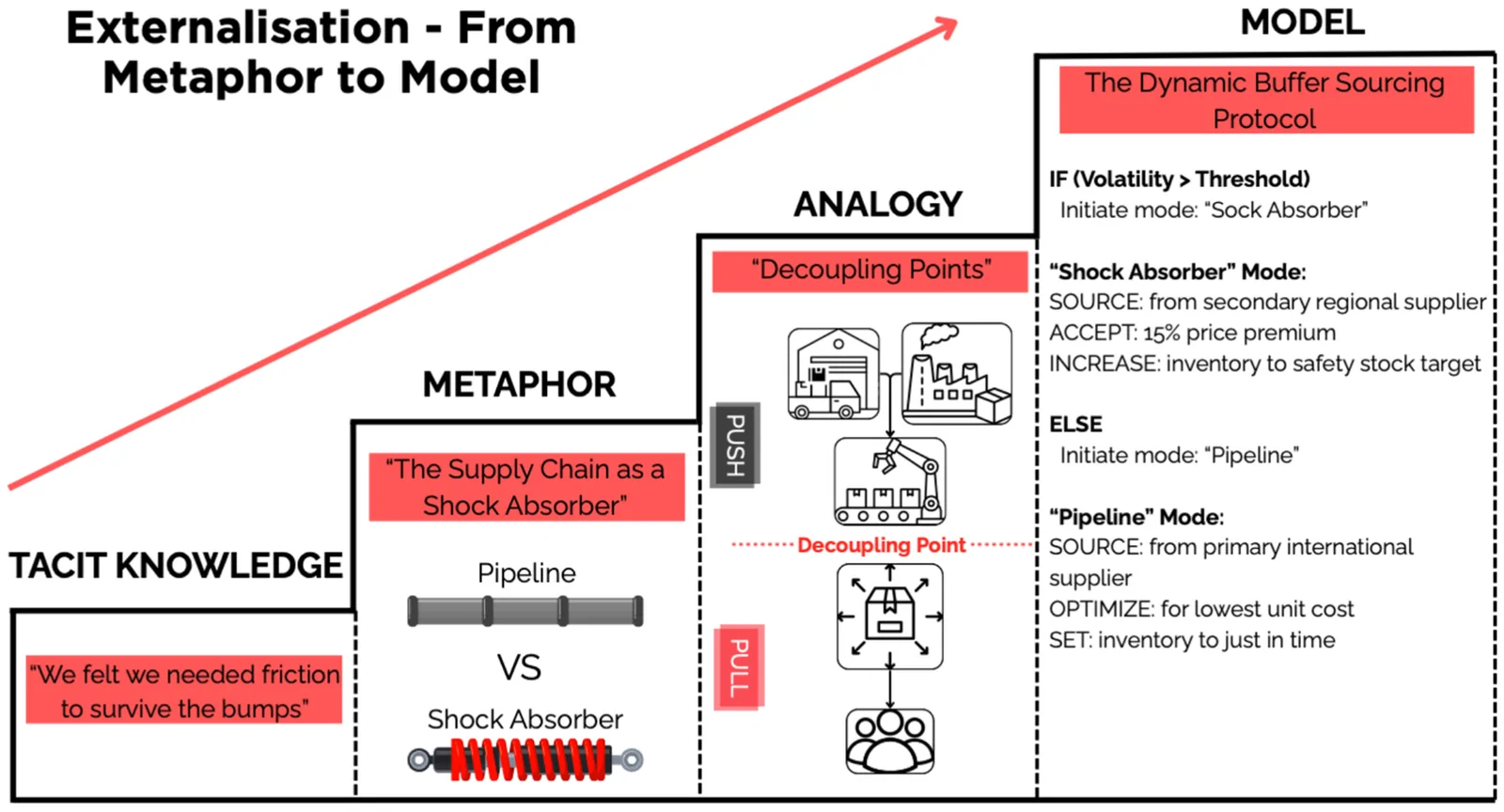
Externalization phase of SECI model applied to case study scenario.

During this dialogue, the team had to select a pathway to codify the solution. They chose the Externalization phase of Nonaka’s SECI model since the specific solution relied heavily on human judgment, social interaction, and interpretive knowledge [2]. Due to the difficulties of verbalizing tacit insights with immediate technical precision, the SECI model guides the practitioner and the organization through a natural cognitive progression to create an explicit, shareable conceptual artifact. This process is visualized in Fig. 12 and the generalization process unfolded in three steps.

**Metaphor:** The team described the new supply chain not as a "pipeline" (the old efficiency view) but as a "shock absorber." This metaphor captured the conflicting ideas of efficiency versus resistance; just as a shock absorber uses friction (inefficiency) to smooth out the ride, the supply chain needed "inefficient" buffers to smooth out geopolitical variance.

**Analogy:** They resolved the contradictions in the metaphor by drawing an analogy to Decoupling Points in systems engineering. This allowed them to distinguish between areas where flow must be rigid and areas where it must be flexible.

**Model:** Finally, this was crystallized into a transferable explicit model: The Dynamic Buffer Sourcing Protocol. This protocol specifies that when external volatility indices exceed a specific threshold, procurement logic must automatically switch from "Just-in-Time" efficiency to "Buffered" redundancy. This step transformed a one-time crisis response into a reusable asset.

### Beta Loop – generalizing the problem boundary conditions

A generalized solution is only effective if the organization knows exactly when to apply it. Therefore, the dialogue must also systematically map the ambient boundary conditions of the problem landscape. If the "Dynamic Buffer" model is misapplied, it could disrupt existing efficient operations, destroying strategic fit.

**Fig. 13:**
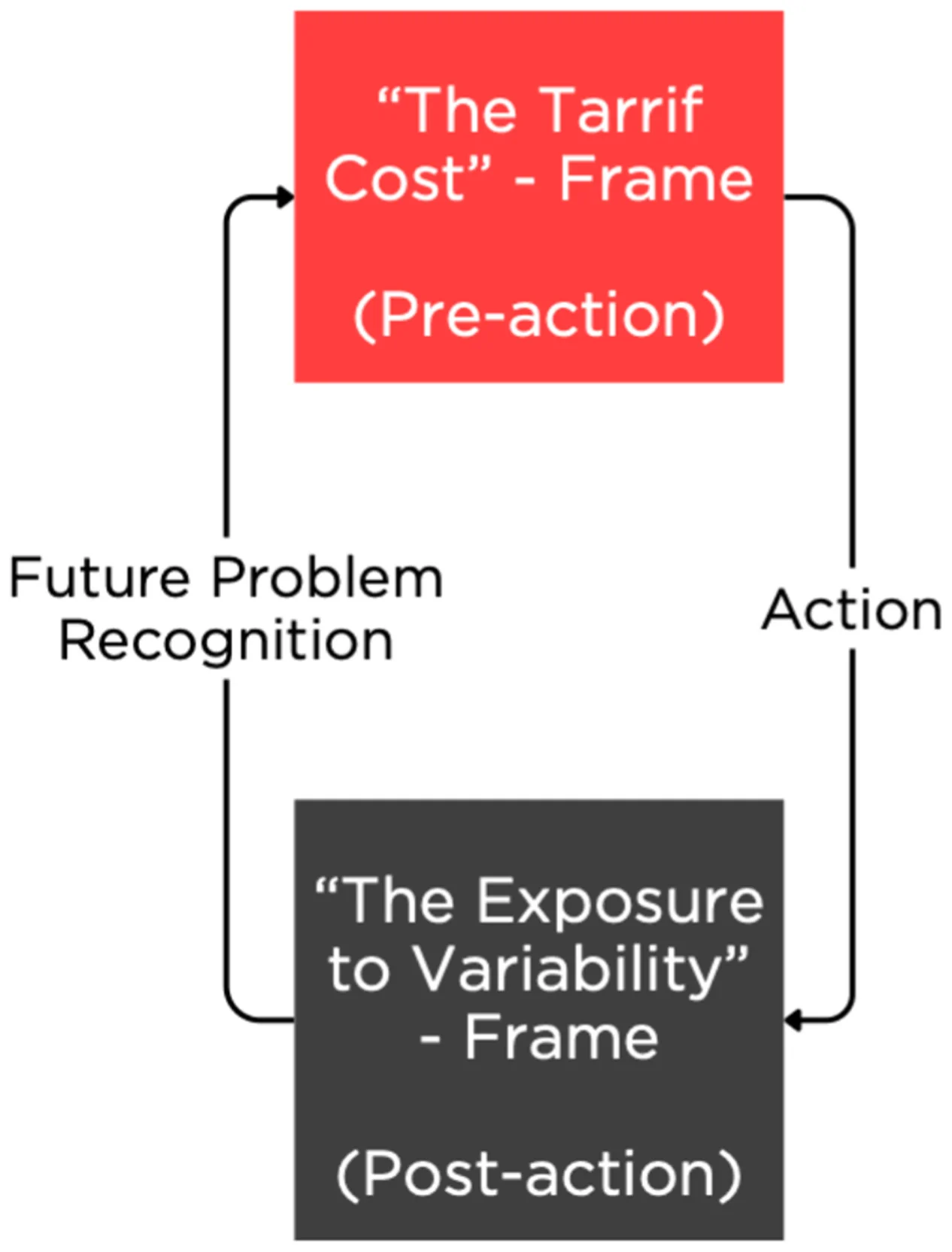
Problem reframing applied to the case study scenario.

To establish these boundaries, the team utilized Schön’s concept of *Problem Framing* [42] to engage in *reflection-on-action*, shown in Fig. 13*.* Action often reveals that the initial diagnosis of a crisis was merely a superficial symptom masking the true structural cause. Initially, the organization had framed the crisis strictly as a "Tariff Cost Issue".

Through their collaborative reflection, the practitioner helps the organization recognize that the cost increase was just a symptom, and the true issue was structural. They systematically reconstruct and reframe the General Problem as "Exposure to Uncontrollable Interface Variability".

This reframing is critical for the organization's future strategic fit. It sets a strict boundary condition ensuring that future managers will not wastefully trigger the expensive "Shock Absorber" protocol for routine price fluctuations, but only when external instability actively threatens operational continuity. By completing this dialogue and rigidly defining the problem structure, the organization successfully transitions the threat from an unpredictable crisis into a defined problem class matched with a verified, reusable standard operating procedure.

### Cross-organizational communication gateway

Before the codified insights can be deployed as organizational knowledge, the practitioner must systematically evaluate the artifact against the five diagnostic questions of the Cross-Organizational Communication Gateway. By subjecting the "Dynamic Buffer Sourcing Protocol" to this gateway, the practitioner provides the following answers to confirm its communication readiness:

1. **Semantic Decoupling and Comprehensibility:** Is the artifact stripped of highly localized acronyms, team-specific jargon, and context-dependent noise so that a practitioner in an entirely different business unit can immediately comprehend its core logic?
  **Yes.** The "Dynamic Buffer Sourcing Protocol" replaces localized references to the original foreign tariff shock with the generalized concept of an "inefficient" shock absorber, ensuring its core logic can be easily comprehended across different business units.
2. **Architectural Scannability and "Clonability":** Is the General Solution structured as an open, modular template (such as an adaptable routine or design pattern) that future teams can quickly scan, clone, and customize without needing to consult the original authors?
  **Yes.** Rather than a rigid step-by-step manual, the protocol functions as an adaptable template, allowing future practitioners to quickly clone the underlying logic of switching from "Just-in-Time" to "Buffered" redundancy based on their own specific volatility indices.
3. **Operational Findability and Metadata Alignment:** Has the artifact been assigned clear, standardized problem typologies and contextual tags so that it can be instantly retrieved when a future practitioner searches the repository during a crisis?
  **Yes.** By abstracting the specific problem into the structural typology of "Exposure to Uncontrollable Interface Variability," the artifact is properly tagged and aligned so it can be instantly retrieved during a crisis.
4. **Absorptive Capacity and Training Readiness:** Does the broader ACTIVE organization possess the requisite baseline knowledge (absorptive capacity) to safely interpret and deploy this protocol, or does its distribution require targeted, high-velocity briefing mechanisms?
  **Yes.** The organization's teams are already familiar with baseline engineering concepts like Decoupling Points and Just-in-Time principles, providing them with the necessary absorptive capacity to safely interpret and deploy the new protocol without requiring extensive training.
5. **Coevolutionary Feedback Mechanisms:** Does the logged asset contain explicit, built-in loops that prompt future practitioners to log their own localized variations, performance metrics, and adjustments upon reuse?
  **Yes.** The protocol is structured as a living document that prompts future practitioners to log their specific threshold adjustments and localized variations upon reuse, establishing a continuous coevolutionary feedback loop.

### Organizational knowledge and managing the double-loop lifecycle

Having passed the Cross-Organizational Communication Gateway, top leadership formally authorizes the artifact's publication. To complete the lifecycle, the host organization acts as the permanent structural home that prevents newly codified insights from evaporating once the immediate crisis passes or personnel depart. The organization's critical role is to systematically archive this data class in its accessible knowledge management database and actively distribute the findings. By formally logging the "Dynamic Buffer Sourcing Protocol" and its boundary conditions into this repository, the organization operationalizes dynamic capabilities, converting an immediate operational response into a long-term resource for sensing, seizing, and transforming.

**Fig. 14:**
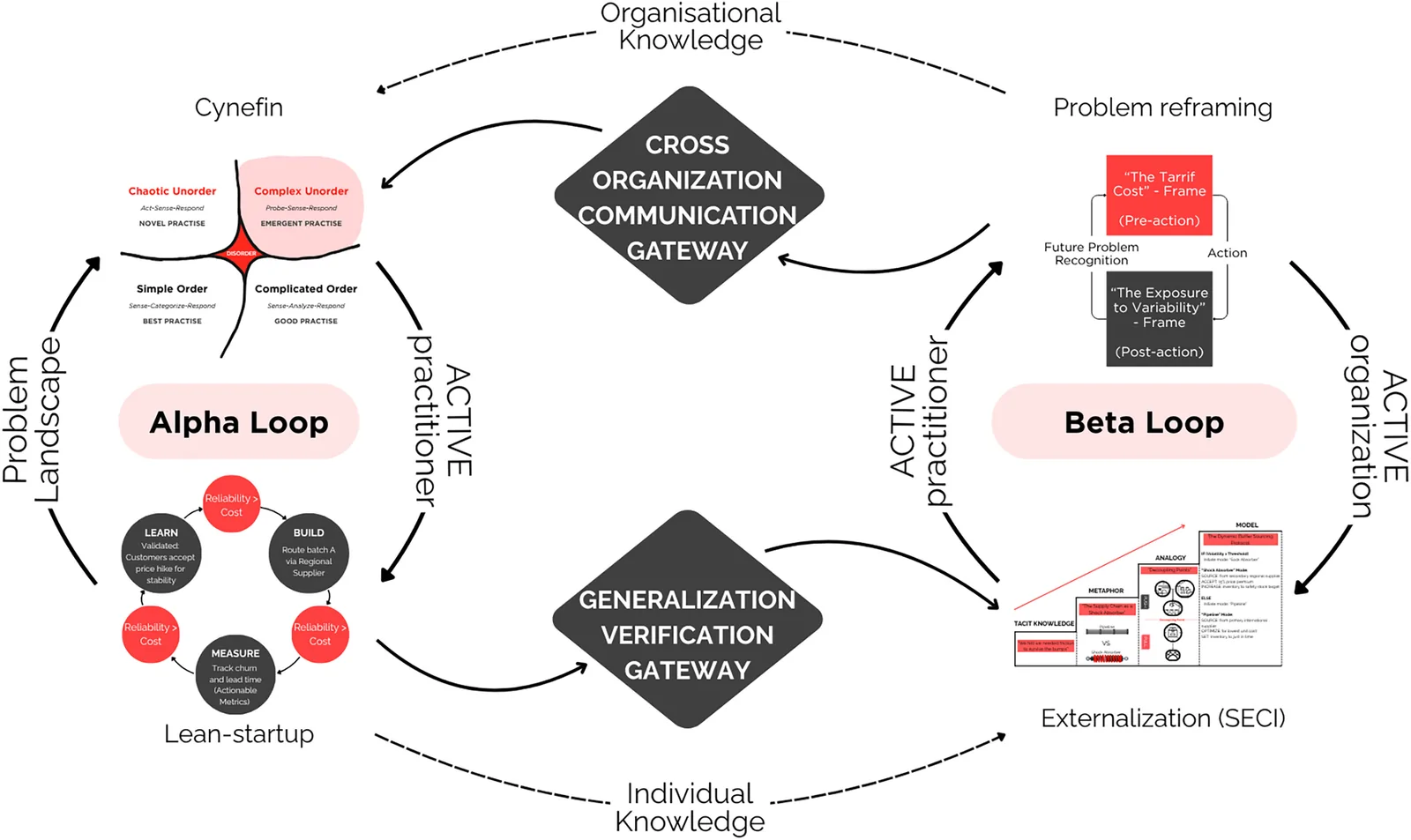
Application of ACTIVE across the illustrative case study scenario.

Going forward, the management of the double-loop cycle is officially established, as traced in Fig. 14. When future practitioners encounter a new crisis within the fluid problem landscape, their first step is to execute an inward sensing search of this central repository. Instead of treating every complex disruption as a novel event, they can instantly retrieve the verified blueprint to stabilize uncertainties without engaging in redundant experimentation. By subsequently adjusting the generalized protocol to fit their unique operational reality and logging those variations, practitioners continuously close the macro-learning loop of the ACTIVE organization, effectively converting individualized tacit knowledge into institutionalized, dynamic capabilities capable of absorbing persistent environmental shocks.

### Method validation and verification

To establish a rigorous architectural foundation for the ACTIVE methodology, a distinction must be made between validation and verification. Drawing on established design science research methodology, validation addresses whether the research is doing the right thing, while verification ensures it is doing things right [49]. Validation interrogates if the problem framing, contextual boundaries, and stakeholder needs are legitimate. Verification interrogates that a proposed solution or method is implemented correctly and is structurally sound. This distinction applies both to the evaluation of the ACTIVE meta-methodology itself, and to how the ACTIVE practitioner evaluates solutions within the problem landscape while using ACTIVE.

### Validation and verification of ACTIVE as a meta-methodology

Validating a meta-methodology designed for highly uncertain, Complex problem landscapes cannot rely solely on formal mathematical proofs, as absolute objectivity is often unattainable when addressing dynamic organizational problems. Instead, according to the relativistic epistemology of the Validation Square [50], validation in design research is a progressive process of "building confidence in its usefulness with respect to a purpose".

ACTIVE achieves foundational credibility through what the Validation Square defines as Theoretical Structural Validity (TSV) [50]. TSV requires accepting both the validity of the individual constructs used within the method and the internal consistency of how those constructs are integrated. ACTIVE fulfills the first requirement because its baseline tools (Design Thinking, the Lean Startup methodology, Adaptive Management, Recognition-Primed Decision Making, and the SECI model) are individually accepted and independently verified across diverse domains. It fulfills the second requirement through its cohesive double-loop architecture and explicit checkpoints, such as the Generalization Verification Gateway, which provides the logical internal consistency connecting these disparate tools.

This theoretical validation strategy is further supported by the Framework for Evaluation in Design Science (FEDS) [51]. FEDS accommodates Ex Ante Artificial/Theoretical Evaluation for socio-technical artifacts and meta-frameworks that guide human behavior. Under the FEDS paradigm, because situational and dynamic methods must be assembled to tackle high-entropy problem landscapes, their primary validity is established through the logical integration of their underlying building blocks [51]. By structurally binding together pre-existing, empirically grounded domain knowledge, ACTIVE's design logic is theoretically validated without introducing unverified, novel technical techniques.

While theoretical validation confirms the relevance of the selected baseline tools, the verification of the ACTIVE meta-method itself focuses strictly on its internal architectural consistency. According to the epistemology of the Validation Square, verifying a method means proving that the flow of information between its distinct constructs is logically sound [50]. For ACTIVE, this structural verification is achieved by mapping the transition from the action-driven Alpha Loop to the generalized Beta Loop, ensuring that the experiential data and specific solutions generated during an intervention serve as adequate, valid inputs for the Generalization Verification Gateway. By structurally verifying that no redundant information is generated and no invalid assumptions are relied upon during this knowledge transfer, the meta-method proves its internal consistency.

### Empirical validity, formative evaluation, and case study boundaries

While ACTIVE possesses TSV, establishing its empirical usefulness requires situational testing. Within the Validation Square, this moves the evaluation toward Empirical Structural Validity (ESV), demonstrating the appropriateness of an example problem to verify the method, as well as Empirical Performance Validity (EPV), which demonstrates that the method is useful for that specific problem [50].

A key boundary of this manuscript is that the manufacturing plant case study serves strictly to demonstrate ESV and EPV as an illustrative scenario, rather than as a universal empirical proof. Relying on a single case study is a recognized limitation; however, within the FEDS framework, this case study functions as a highly appropriate formative, artificial evaluation episode [51]. FEDS dictates that evaluation should progress strategically. Since ACTIVE addresses situations with high uncertainty, early formative evaluations are necessary to assess the operational mechanics and theoretical pathways of the method before progressing to highly resource-intensive, naturalistic, and summative field evaluations.

Without extensive multi-site longitudinal research to confirm full Theoretical Performance Validity, or general usefulness beyond the example problem [50], claims regarding the reproducibility of the ACTIVE protocol must be interpreted cautiously.

### Verification and validation within the active lifecycle

When actively applying the methodology in the field, the ACTIVE practitioner must also navigate the distinct phases of verification and validation to ensure that localized interventions are successfully converted into reusable organizational assets. Within the ACTIVE architecture, verification is primarily addressed through the Generalization Verification Gateway and the Cross-Organizational Communication Gateway. Verification ensures that the proposed solution is implemented correctly and is structurally sound [49].

After the practitioner has engaged with the problem landscape, the Generalization Verification Gateway acts as the first operational checkpoint to verify if the specific solution contains stable, generalizable principles. Verification at this stage confirms that the tactical action is structurally sound enough to be generalized into a General Solution without cluttering organizational memory.

Once the General Solution and its boundary conditions have been codified in the Beta Loop, the Cross-Organizational Communication Gateway provides the second verification checkpoint. Its diagnostic criteria are set out in Table 7 and are not repeated here. In verification terms, its role is narrower than that of the first gateway. It confirms only whether the resulting explicit artifact is sufficiently understandable, accessible, and decoupled from localized jargon for the rest of the ACTIVE organization to reference and implement without translation by the original authors. Artifacts that fail these benchmarks return to the Beta Loop for formatting refinement to protect the corporate repository from information bloat and low-fidelity documentation.

While these dual gateways handle verification, validation operates on a broader scale. Validation asks whether the original problem framing and the contextual boundaries were legitimate and well-founded for the stakeholders involved [49]. Crucially, because true validation requires confirming relevance and effectiveness in a naturalistic, real-world problem landscape, it cannot be definitively claimed after the first instance of ACTIVE being applied. Passing the verification gateway only proves that a localized tactical action was structurally consistent. Under both FEDS and the Validation Square [50,51], achieving deep summative naturalistic validation and Theoretical Performance Validity requires repeated application.

True validation occurs only when a future ACTIVE practitioner retrieves a previous General Solution and reapplies it to a new specific problem. By executing it again in a new context, the organization tests the transferability of the knowledge, clearly noting if the solution's boundary conditions hold. Therefore, ACTIVE functions as an ongoing learning system where validation is a continuous, cyclical process of retrieving, applying, and refining knowledge across successive environmental shocks.

## Ethics statement

The authors confirm that the work described in this article is original and has not been published previously, except in the form of a preprint, abstract, published lecture, academic thesis, or registered report, in accordance with Elsevier’s policy on multiple, redundant, or concurrent publication. The article is not under consideration for publication elsewhere, and its submission has been approved by all authors as well as by the responsible authorities at the institution where the work was carried out. If accepted for publication, this article will not be published in the same form, in English or in any other language, including electronically, without the prior written consent of the copyright holder.

## CRediT author statement

**Reeder Vermaak:** Conceptualization, Investigation, Writing - Original Draft, Visualization.

**Meelan Roopa**: Conceptualization, Resources, Writing - Review & Editing, Supervision.

**Sara Grobbelaar:** Writing - Review & Editing, Supervision.

## Declaration of generative AI and AI-assisted technologies in the writing process

During the preparation of this manuscript, the authors used OpenAI’s ChatGPT (GPT-5) to assist with language refinement and readability, and Google NotebookLM to support source-consistency checks. All AI-assisted outputs were reviewed and edited by the authors as necessary. The authors take full responsibility for the content and integrity of the publication.

## Declaration of competing interest

The authors declare that they have no known competing financial interests or personal relationships that could have appeared to influence the work reported in this paper.

## Figures and Tables

**Figure:**
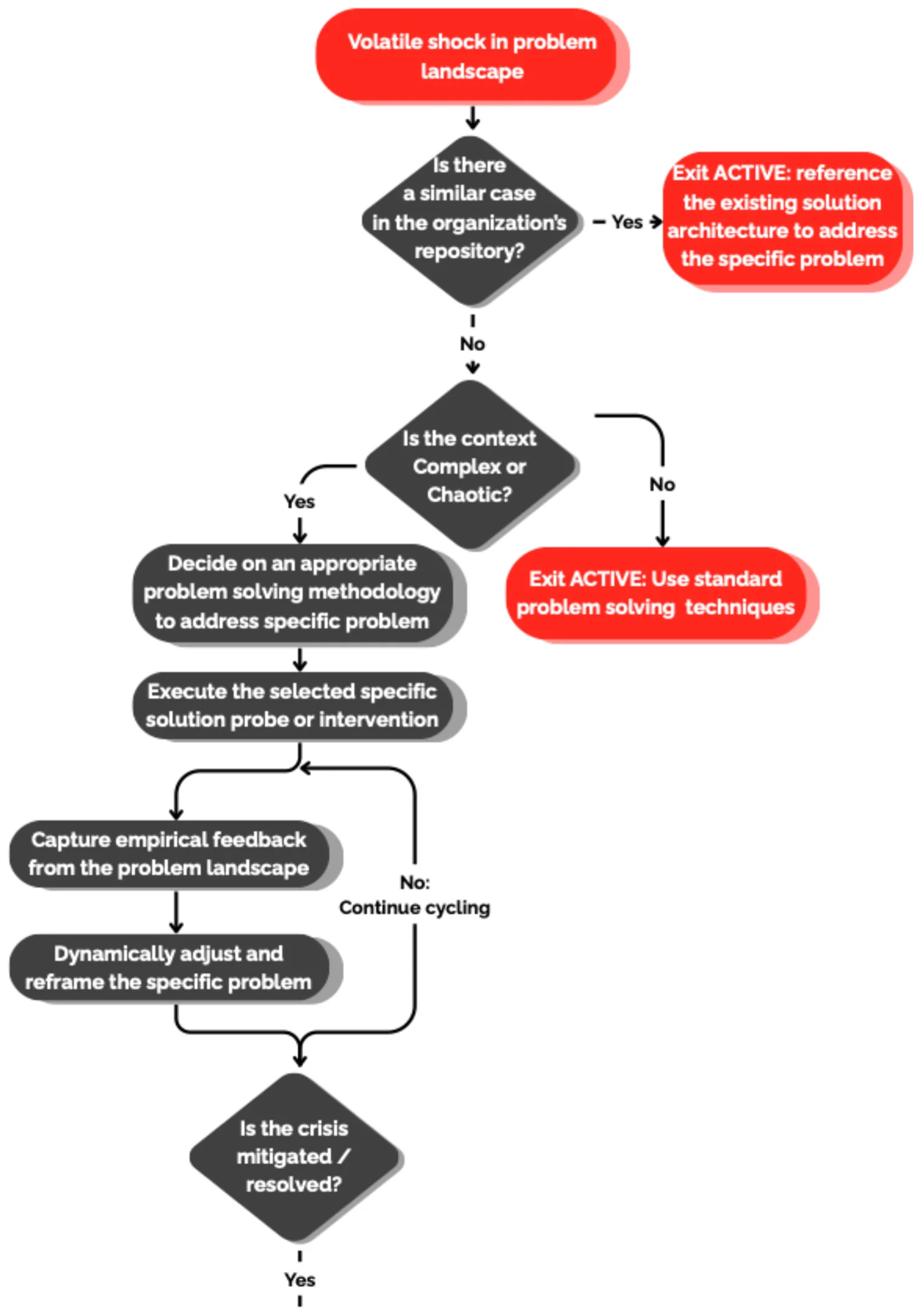

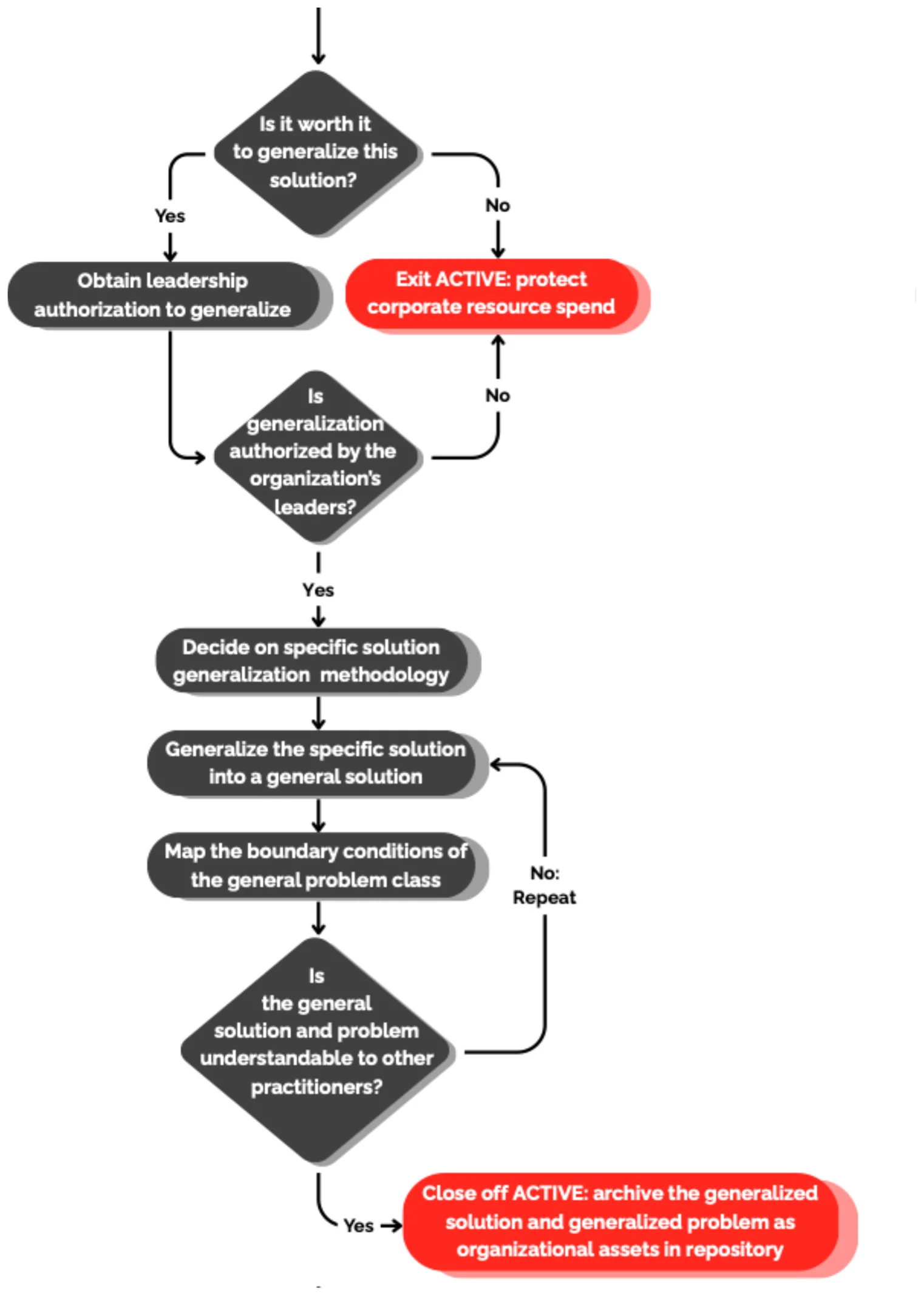


## Data Availability

No data was used for the research described in the article.
